# Human Monocytic Suppressive Cells Promote Replication of *Mycobacterium tuberculosis* and Alter Stability of *in vitro* Generated Granulomas

**DOI:** 10.3389/fimmu.2018.02417

**Published:** 2018-10-23

**Authors:** Neha Agrawal, Ioana Streata, Gang Pei, January Weiner, Leigh Kotze, Silke Bandermann, Laura Lozza, Gerhard Walzl, Nelita du Plessis, Mihai Ioana, Stefan H. E. Kaufmann, Anca Dorhoi

**Affiliations:** ^1^Max Planck Institute for Infection Biology, Department of Immunology, Berlin, Germany; ^2^University of Medicine and Pharmacy Craiova, Human Genomics Laboratory, Craiova, Romania; ^3^Division of Molecular Biology and Human Genetics, Department of Biomedical Sciences, Faculty of Medicine and Health Sciences, SAMRC Centre for Tuberculosis Research, DST and NRF Centre of Excellence for Biomedical TB Research, Stellenbosch University, Tygerberg, South Africa; ^4^Institute of Immunology, Federal Research Institute for Animal Health, Friedrich-Loeffler-Institut, Insel Riems, Germany; ^5^Faculty of Mathematics and Natural Sciences, University of Greifswald, Greifswald, Germany

**Keywords:** tuberculosis, *Mycobacterium tuberculosis*, myeloid-derived suppressor cells, granuloma, IL-10, PD-L1

## Abstract

Tuberculosis (TB) has tremendous public health relevance. It most frequently affects the lung and is characterized by the development of unique tissue lesions, termed granulomas. These lesions encompass various immune populations, with macrophages being most extensively investigated. Myeloid derived suppressor cells (MDSCs) have been recently identified in TB patients, both in the circulation and at the site of infection, however their interactions with *Mycobacterium tuberculosis* (*Mtb*) and their impact on granulomas remain undefined. We generated human monocytic MDSCs and observed that their suppressive capacities are retained upon *Mtb* infection. We employed an *in vitro* granuloma model, which mimics human TB lesions to some extent, with the aim of analyzing the roles of MDSCs within granulomas. MDSCs altered the structure of and affected bacterial containment within granuloma-like structures. These effects were partly controlled through highly abundant secreted IL-10. Compared to macrophages, MDSCs activated primarily the NF-κB and MAPK pathways and the latter largely contributed to the release of IL-10 and replication of bacteria within *in vitro* generated granulomas. Moreover, MDSCs upregulated PD-L1 and suppressed proliferation of lymphocytes, albeit with negligible effects on *Mtb* replication. Further comprehensive characterization of MDSCs in TB will contribute to a better understanding of disease pathogenesis and facilitate the design of novel immune-based interventions for this deadly infection.

## Introduction

*Mycobacterium tuberculosis* (*Mtb*) is the causative agent of tuberculosis (TB), a disease that accounted for 1.7 million deaths in 2016 (1). Caseous granulomas which develop at the site of *Mtb* infection are considered a hallmark of pulmonary TB (2). Albeit specific for TB, these lesions are not pathognomonic, granulomas are triggered also by unrelated bacteria, fungi and parasites as well as by foreign bodies (3). The cellular composition of TB granulomas may vary with disease stage. Generally, lesions consist of macrophages, lymphocytes and transformed macrophages, including epithelioid and multinucleated giant cells as well as foamy macrophages (4, 5). Trajectories and the fate of granulomas are determined by a plethora of secreted factors, such as cytokines and eicosanoids, which are locally produced by immune cells (6), *in situ* changes in cellular composition, as well as viability, replicative and metabolic features of the mycobacteria (7, 8). Balanced abundances of the pro-inflammatory cytokines IFN-γ and TNF-α are associated with bacterial clearance while regulatory cytokines like IL-10 offer limited protection to TB (2, 9, 10). Presence of selected immune cell subsets, their location, as well as their propensity to produce soluble mediators thus control stability of granulomas and TB progression.

Despite recent new insights into mechanisms governing *Mtb* interaction with immune cells, understanding of factors controlling *Mtb* survival within pulmonary TB granulomas, specifically in human lesions remains poorly defined (7). The diversity and the activation spectra of immune cells present within granulomas are currently acknowledged (11, 12). Yet, how newly described subsets imprint on granuloma stability and *Mtb* replication remains to be established. Myeloid-derived suppressor sells (MDSCs) have been recently detected in pleural effusion and in the peripheral blood in TB patients (13–15). MDSCs encompass heterogeneous myeloid cells, both monocytic- and neutrophil-like, which suppress T-cell immunity through high expression of arginase-1, inducible nitric oxide synthase, indoleamine dioxygenase, cyclooxygenase, IL-10 or reactive oxygen species (16). In murine models, MDSCs harbor mycobacteria, promote tissue damage and their depletion alone or in combination with canonical TB chemotherapy lowers bacillary burdens and improves pathology (17–21). These studies have identified MDSCs within the lungs and highlighted their capacity to alter or directly produce and respond to cytokines critical for granuloma stability, notably IFN-γ, TNF-α, IL-10, and IL-6 (13–15, 17–23). Moreover, investigations performed in the non-human primate model report populations of macrophages co-expressing nitric oxide synthase and arginase-1 (24). Such cells resemble MDSCs and were detected specifically in necrotic granulomas in macaques. The interactions of human MDSCs with *Mtb* including their ability to modulate granuloma-like structures have not been addressed so far.

Murine models represent valuable tools to study host-mycobacteria interactions (25). However, the extent of similarity between disease pathophysiology and lung lesions in murine TB and human patients varies with the murine model utilized (26). Particularly TB granulomas are hardly reproduced by TB mouse lung lesions. To overcome such experimental limitations many investigators have independently developed and characterized *in vitro* granuloma models (27–38). Such structures enable the study of human cell-cell interactions upon mycobacterial infection and thereby early events in TB. Absence of unique lung environment and lack of fibrosis, encapsulation and caseation represent major limitations of such models. However, these structures mimic human TB granulomas especially regarding the cellular composition. *In vitro* granulomas contain epithelioid cells, foamy macrophages and multinucleated giant cells, along with other immune cells usually observed in TB lesions (32). Considering the limitations of this model, we termed such *in vitro* generated multicellular aggregates, “*in vitro* granuloma like structures” (IVGLSs). We investigated the functions of human monocytic MDSCs in TB by characterizing their responses to mycobacteria and using a well-defined *in vitro* granuloma model (35). We observed that MDSCs support *Mtb* replication within IVGLSs and identified molecular requirements and signaling pathways operative in MDSCs and driving such effects.

## Materials and methods

### Isolation and culture of cells

The buffy coats were obtained from healthy donors through the blood bank of German Red Cross (Deutsches Rotes Kreuz, DRK). Donors were kept anonymous and their latent TB status was unknown. Peripheral blood mononuclear cells (PBMCs) were isolated from buffy coats by density gradient centrifugation (Biocoll, Biochrom GmbH, DE). After thorough washing with phosphate buffer saline (PBS), mononuclear cells were cultured, cryopreserved or further processed according to the requirement of each experiment.

Generation of human MDSCs was done as previously described (39). Briefly, CD14^+^ cells were isolated from the PBMC fraction by positive magnetic bead isolation according to manufacturer's instructions (Miltenyi Biotech, DE). 5 × 10^6^ monocytes were cultured in untreated Petri dishes in 10 ml IMDM (Gibco, IRL) supplemented with heat inactivated 10% (v/v) fetal calf serum (FCS) (Lonza Group, CH), 1% L-glutamine, 1% HEPES and 0.1% β-mercaptoethanol (β-ME) (Gibco, IRL), recombinant IL-4 and GM-CSF (both at 1000 U/ml; PeproTech, NJ, USA) and PGE_2_ (1 μM; Sigma-Aldrich, MO, USA). Cell were differentiated for 6 days at 37°C and 5% CO_2_. This protocol resulted in generation of loosely attached MDSCs (39). In selected experiments, for comparative analysis cells were differentiated in presence of human AB clotted serum (Sigma-Aldrich, MO, USA), herein abbreviated hsMDSCs.

To generate monocyte-derived macrophages (MDMs), 2 × 10^7^ PBMCs were suspended in 10 ml PBS and incubated for 1 h in 75 cm^2^ flask at 37°C, 5% CO_2_. Afterwards cells were washed with PBS and incubated with 20 ml RPMI-1640 supplemented with 1% L-glutamine, 1% HEPES, 0.1% β-ME and heat inactivated 10% human AB clotted serum. Flasks were incubated for 6 days at 37°C, 5% CO_2_. Harvesting of MDMs was done using accutase (Gibco, IRL).

Whole PBMCs were cryopreserved in 10% dimethyl sulfoxide (DMSO) in FCS at the day of isolation for further co-cultures with IVGLSs obtained from matched donors in granuloma studies. Similarly, CD14^−^ cells or PBMCs were cryopreserved from the matched donors for purification of untouched T-cells using naïve pan T-cell isolation kit (Miltenyi Biotech, DE) according to vendor's instructions for further co-culture studies.

Isolation of MDSCs from active TB patient samples was done as described previously (23). In brief, PBMCs were isolated from blood within 2 h of collection using density gradient centrifugation. Unlabeled HLADR^−^ cells were isolated using HLADR microbeads (Miltenyi Biotech, DE) and subjected to isolation of CD33^+^ cells using CD33^+^ microbeads (Miltenyi Biotech, DE) according to manufacturer's instructions. Cells were cultured in RPMI-1640 (Biowest, France) supplemented with 1% L-glutamine and 2% FCS. Donors were negative for HIV and positive for drug susceptible TB.

Cell viabilities were estimated after various assays using the LDH cytotoxicity kit (Roche Holding AG, CH).

### Bacterial culture and infection of MDSCs and MDMs

*M. tuberculosis* laboratory strain H37Rv and *M. bovis* BCG cultures were grown to early-log phase at 37°C in Middlebrook 7H9 broth (Thermo-Fischer Scientific, NH, USA) supplemented with 10% albumin dextrose catalase (Thermo-Fisher Scientific, MA, USA), 0.05% glycerol and Tween 80 (Sigma-Aldrich, MO, USA). Bacilli were used within four passages after thawing and were harvested when cultures reached an optical density (at 600 nm) of 0.4 to 0.6. For infection, bacteria were washed with PBS and passed through 27G needle to obtain a single cell suspension. MDSCs and MDMs were infected at the multiplicity of infection (MOI) 10 and 20 for 4 h in rotation at 37°C to minimize loss of cells due to poor adherence and to maintain similar conditions for both cell types (17). The MOI 10 resulted in 1 to 2 bacteria per cell at 4 h post infection (data not shown). MOI 20 was used only for short course experiments.

### Flow cytometry

Cells were blocked with human Fc receptor (Miltenyi Biotech, DE), stained and fixed with 4% paraformaldehyde (PFA) before proceeding for acquisition. For each donor and each condition, three technical replicates were processed. Following antibodies were used: CD3 (clone UCHT1), CD8 (clone HIT8a), CD11b (clone ICRF44), CD33 (clone WM53), CD14 (clone M5E2), CD15 (clone H198), Lineage cocktail (CD3, CD19, CD20 and CD56; clones UCHT1, HIB19, 2H7 and 5.1H11, respectively), Ki-67 (clone Ki-67) and PD-L1 (clone 29E.2A3) from BioLegend, CA, USA; CD4 (clone RPA-T4), HLA-DR (clone G46-6) and AnnexinV from BD Biosciences, NJ, USA; live/dead staining kit from Thermo-Fisher Scientific, MA, USA. In selected studies, MDSCs, MDMs or T cells were labeled with 5 μM CFSE (CellTrace, Invitrogen, CA, USA) following manufacturer's instructions. All samples were acquired in the BSL3 facility on a CytoFLEX flow cytometer (Beckman Coulter, CA, USA).

### T-cell suppression assay

[^3^H]-Thymidine incorporation assay was employed to assess proliferation of T-cells in the BCG studies and the CFSE dilution method as well as the Ki-67 staining were used for the *Mtb* investigations. Each donor and conditions were studied in triplicate. Lymphocytes were polyclonally activated in UV sterilized flat bottom 96-well Maxisorb plates which were coated with 1 μg/ml of anti-CD3 (clone UCHT1) and anti-CD28 (clone 37407) (R&D Systems, MN, USA) antibodies in PBS. Unstimulated T-cells served as negative control and stimulated T-cells without addition of the MDSCs were used as positive control. T-cells were co-cultured with varying amounts of autologous naïve or BCG-infected MDSCs (MOI 10). Plates were sealed with parafilm and incubated at 37°C and 5% CO_2_. After 48 h of co-culture, 1μCi of [^3^H]-Thymidine was added and plates were incubated for additional 18 h. Plates were either directly read or stored at −20°C for further harvesting. FilterMate Harvester was used to quantify counts per minute (CPM) using a Microplate Plate Counter (PerkinElmer, MA, USA). In separate assays, cells were also stained with 7AAD and AnnexinV to estimate numbers of live T-cells upon co-culture. *Mtb* infected MDSCs were co-cultured in BSL3 laboratory with autologous T-cells after prior labeling of the lymphocytes with CFSE. Similar to BCG infection, MDSCs were infected with H37Rv at MOI 10 for 4 h and added to stimulated T-cells in various ratios. After 72 h of incubation at 37°C and 5% CO_2_, cells were isolated and processed for further assessment of the proliferation by staining for phenotype and proliferation markers (CD3, CD4, and CD8; Ki67) and analyzed by flow cytometry.

### Generation of IVGLSs

The protocol for the generation of IVGLSs was adapted from Agrawal et al. (35). In brief, 4 × 10^5^ PBMCs were seeded on the day of PBMC isolation in 48 well tissue culture treated plates. Cells were incubated overnight in 10% FCS supplemented RPMI GlutaMAX media (Thermo-Fisher Scientific, MA, USA). The next day cells were infected with H37Rv at an MOI of 0.005 (calculated according to the total number of PBMCs cultured per well, that is approximately 2,000 bacteria per well) followed by incubation for 4 h and subsequent gentamicin (100 μg/ml) treatment for 2 h to remove extracellular bacteria. Afterwards, plates were centrifuged at 100 × g for 3 min, media were replaced with fresh 500 μl media per well and plates were incubated until further testing. To assess the effect of different cell types on IVGLSs; (a) MDSCs, (b) MDMs (both after 6 days of differentiation) and (c) PBMCs (cryopreserved) were collected, washed and added to running IVGLSs cultures at day 5 post induction. Distinct ratios of MDSCs/MDMs/PBMCs compared to the number of PBMCs employed for generation of IVGLSs were used. Therefore, ratios 1:2 and 1:4 correspond to addition of 2 × 10^5^ or 1 × 10^5^ cells at day 5 post induction, respectively, where 4 × 10^5^ PBMCs were seeded to induce IVGLSs at day 0. In all of these co-culture studies MDSCs/MDMs/PBMCs were kept uninfected while adding carefully to IVGLSs. Any further readouts were done after 48 h of co-cultures, that is day 7 post generation of the IVGLSs.

### Quantification and imaging of the IVGLSs

IVGLS plates were centrifuged at 100 × g for 3 min, supernatants were removed and cells were fixed overnight in 4% PFA in PBS. Next day PFA was washed away and cells were stained with DAPI. Images were acquired on the high content platform ArrayScan XTI (Thermo-Fisher Scientific, MA, USA) and subsequently analyzed using an in-built software, HCS Studio (Thermo-Fisher Scientific, MA, USA). HCS analysis protocol was set to facilitate identification of the individual objects, notably granuloma-like structures, and to reject background noise. Reported valid granulomas were equal or larger than 400 μm^2^, which was lowest detection limit of the software for high specificity according to our protocol. The same acquisition settings were applied to all investigated samples. Each condition was acquired from triplicate wells for each donor. IVGLSs were in addition induced on Poly-L-Lysine coated coverslips to observe these structures, with or without addition of MDSCs/MDMs, using confocal microscopy (Leica TCS SP8; Leica Microsystems, DE). Giemsa staining was performed using standard protocol (35) to observe cellular structures in 48 well plates.

### Estimation of bacterial colony forming units (CFUs) from IVGLSs

Cell culture plates were centrifuged at 100 × g for 3 min and supernatants collected for further analyses. Cells were lysed with 100 μl of 0.1% Triton-X100 in PBST (0.05% Tween80 in PBS). Serial dilution of lysates were plated on Middlebrook 7H11 agar (Fischer Scientific, NH, USA) plates supplemented with 10% oleic albumin dextrose catalase (Thermo-Fisher Scientific, MA, USA) and 0.5% glycerol. Total CFUs were counted after 21 days of incubation at 37°C. For contact dependency assessment, transwells of 0.4 μm pore sized polycarbonate inserts were used. IVGLSs were generated in the lower chamber and MDSCs or MDMs were added with or without inserts to these wells. Samples were processed and plated for CFU enumeration after 48 h of co-cultures.

### Cytokine measurements

Supernatants collected from various samples (monocultures and co-cultures) were passed through 0.2 μm pore size filters and stored at −20°C until used. Commercially available ELISA kits from R&D Systems (MN, USA) were used to detect concentrations of IL-6, IL-10, IFN-γ, and TNF-α according to manufacturer's instructions.

To assess the effect of specific inhibitors on cytokine release, cells were pre-incubated for 1 h with several inhibitors at varying concentrations (1, 5, or 30 μM) and infected with *Mtb* at MOI 10 afterwards. For each cell type and each concentration of inhibitor, internal controls with corresponding amount of DMSO were included. The following inhibitors were used: SB203580 (p38 inhibitor) and PD98059 (MEK1/2 inhibitor) (Cell Signaling, MA, USA); PD0325901 (MEK1/2 inhibitor), BMS345541 (NF-κB inhibitor), FR180204 (ERK1/2 inhibitor), Tanshinone IIa (AP1 inhibitor) and LY294002 (PI3K inhibitor) (Sigma-Aldrich, MO, USA); Wortmanin (PI3K inhibitor) (Merck-Calbiochem, DE) and SR11302 (AP1 inhibitor) (Tocris Biosciences, Bristol, UK). Supernatants were collected after 24 h of culture, filtered and assessed for cytokine concentration.

To observe antigen specific responses, MDSCs purified from TB patients were stimulated either with tuberculin protein purified derivative (PPD) (Statens Serum Institute, Denmark) at 4 μg/ml or only media. According to other experimental conditions, (data not included) co-stimulatory antibodies CD28 (clone L293) and CD49d (clone L25) (BD Biosciences, NJ, USA) were also added to isolated MDSCs at 250 ng/ml. Cytokines were measured from the supernatants after 20 h of culture using multiplex bead based assay (IL-10 by R&D Systems Incorporated, MP, USA and IL-6 by Merck, DE) on a Luminex platform (Bio Rad laboratories, CA, USA). Culture interval also included addition of Brefeldin A (Sigma-Aldrich, MO, USA) before 4 h of completion of assay; to measure intracellular cytokines from same samples (data not included).

### Neutralization assay

Two different neutralization experiments were independently performed: (a) IL-10 and (b) PD-L1. For each assay, after harvesting mature cells, MDSCs or MDMs were pre-incubated with neutralizing antibodies for 30 min, at 10 μg/ml. The following antibody clones were employed: IL-10 (clone 23738) and IL-10Ra (clone 37607) (R&D Systems, MN, USA), PD-L1 (clone MIH1, Thermo-Fisher Scientific, MA, USA), isotype IgG1κ (clone MG1-45), and isotype IgG2bκ (clone MG2b-57) (BioLegend, CA, USA). Afterwards cells were added to day 5 IVGLSs of matched donors and analyzed after 48 hr of co-cultures. Similarly, IVGLSs were treated with different pathway inhibitors (SB203580, PD98059, BMS345541, LY294002, Wortmanin and SR11302), as described in the above section, with or without addition of MDSCs or MDMs at day 5 post induction of IVGLSs. The CFUs were estimated after 48 h of co-culture.

### RNA expression and qRT-PCR

After 4 h of *Mtb* infection, 5 × 10^5^ naïve or infected MDSCs and MDMs were transferred in 1 ml TRIzol reagent (Invitrogen, CA, USA) and kept frozen at −80°C until RNA isolation. Reverse transcription was performed using High-Capacity cDNA Reverse Transcription Kit (Applied Biosystems, CA, USA). qRT-PCR was analyzed on a Biomark HD System (Fluidigm Corporation, CA, USA) with TaqMan Fast universal mix and primers. The following primers were used: *CCL5*: Hs00982282_m1, *CXCL10*: Hs01124251_g1, *IL6*: Hs00174131_m1, *IL10*: Hs00961622_m1, *PDL1*: Hs00204257_m1, *TGFB*: Hs00998133_m1, and *TNFA*: Hs01113624_g1.

### Signal pathway analysis and western blotting

Cells were rested in 1% FCS supplemented RPMI-1640 for 5 h at 37°C in a rolling incubator. Equal numbers of MDSCs and MDMs (5 × 10^5^ cells per condition) were stimulated either with H37Rv (MOI 20) or with LPS (25 ng/ml) for various time intervals ranging from 10 min to 2 h. At every time point cells were centrifuged at 300 × g, 4°C for 5 min followed by two washings with cold PBS and incubation with RIPA buffer (BD Biosciences, NJ, USA) supplemented with phosphatase and protease inhibitor cocktail (Roche Holding AG, CH) at 4°C for 10 min. Supernatants were collected after centrifugation at 16,000 × g for 10 min at 4°C. Samples were denatured at 96°C for 10 min after addition of sample buffer (Sigma-Aldrich, MO, USA) and were either subjected to immediate electrophoresis or stored at −80°C. After electrophoresis and transfer to nitrocellulose membrane, samples were blocked with 5% low fat milk in PBST (0.1% Tween20 in PBS). Membranes were incubated overnight with primary antibodies; NF-κB p65, NF-κB p-p65, p38 MAPK, p-p38 MAPK, ERK1/2, p-ERK1/2, JNK, p-JNK (all from Cell Signaling, MA, USA) at 4°C. Subsequently, membranes were incubated with the HRP-conjugated anti-mouse IgG or anti-rabbit IgG antibodies (Cell Signaling, MA, USA) and developed using ECL detection kit (Thermo-Fisher Scientific, MA, USA).

### Statistical analysis and software

Data were analyzed with GraphPad Prism 7 (Graphpad, USA), FlowJo (FlowJo LLC, OR, USA), HCS Studio (Thermo-Fisher Scientific, MA, USA), Fiji ImageJ (Wayne Rasband, National Institutes of Health, USA), Fluidigm Real-Time PCR analysis software (Fluidigm Corporation, CA, USA), and R statistical programming environment (40). The paired Student's *t*-test was applied to estimate statistical significance for most data sets. For analysis of various treatments on a cell type, within a group, one-way ANOVA with Dunn's multiple comparison was applied. RNA expression data was analyzed by two-way ANOVA followed by Bonferroni correction. Comparison of the distributions of granuloma sizes was performed using a Wilcoxon test comparing the distribution medians of each donor in two groups and confirmed by a randomization test.

## Results

### MDSCs retain their suppressive property upon *Mtb* infection

**Figure 1:**
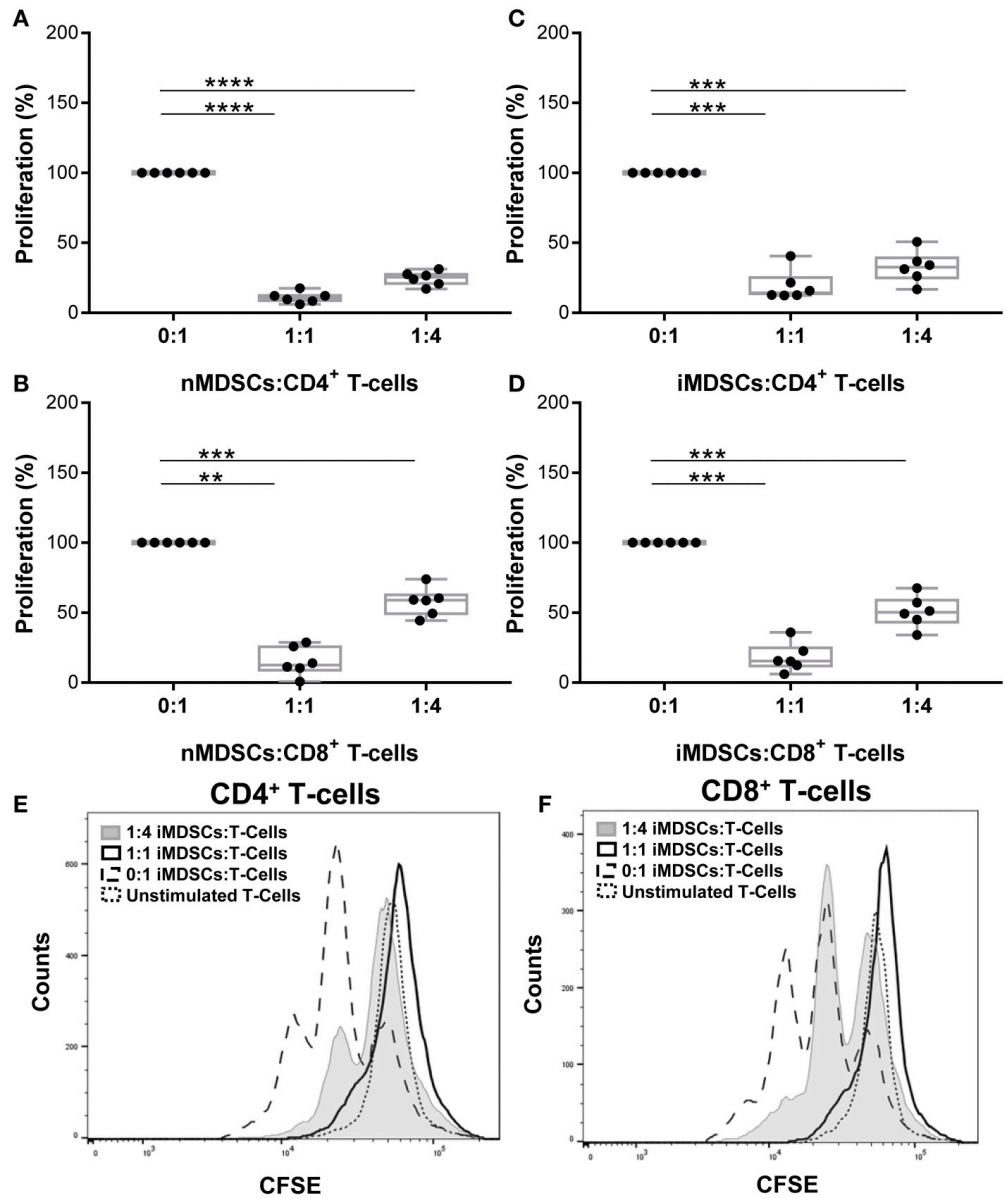
*In vitro* generated human MDSCs maintain suppressive activity following Mtb infection. Proliferation of polyclonally stimulated, CFSE-labeled T-cells co-cultured with naïve (nMDSCs) **(A,B)** and *Mtb*-infected (MOI 10) MDSCs (iMDSCs) **(C,D)** for 72 h. Number of T-cells was kept constant for all conditions with varying amounts of added MDSC; no MDSCs (0:1), 1/4th MDSCs (1:4) and equal MDSCs (1:1). Representative histograms are presented for one donor **(E,F)**. Each symbol corresponds to a single donor, shown are median±IQR. Data are pooled from 2 independent experiments (*n* = 6 individuals); paired Student's *t*-test, ^**^p ≤ 0.01, ^***^p ≤ 0.001, ^****^p ≤ 0.0001.

The phenotype of *in vitro* differentiated MDSCs was examined following the gating strategy suggested by Bronte et al. (16) (Supplementary Figure 1). Cells were lineage negative and expressed high CD33 and CD14. Differentiation of MDSCs with human serum down-regulated CD14 expression and left other cell surface markers unchanged (hsMDSCs; Supplementary Figure 1). We validated the suppressive abilities of *in vitro* generated human MDSCs (Figures 1A,B) and observed that these properties were preserved upon infection with virulent mycobacteria (Figures 1C,D; Supplementary Figure 2). *Mtb*-infected MDSCs suppressed proliferation of CD4^+^ (Figures 1C,E) and CD8^+^ (Figures 1D,F) T-cells upon co-culture. The results of CFSE dilution assay were confirmed by Ki-67 proliferation experiment (Supplementary Figure 3A). Infection with BCG similarly resulted in maintenance of the T-cell suppressive activity of human MDSCs (Supplementary Figure 3B). The suppressive capacity of *Mtb*-infected MDSCs was observed irrespective of the sera used for their differentiation (Supplementary Figure 4A). MDSCs generated with FCS were employed for further analyses, unless otherwise indicated. Altogether, these co-culture studies suggest that the suppressive property of MDSCs is retained upon mycobacterial infection independent of key virulence factors, such as those encoded by region of difference 1 (RD1), which is absent in BCG. Suppression was maintained upon infection implying that MDSCs can affect the cross-talk between T-cells and myeloid cells in TB.

### MDSCs alter the structure of and *Mtb* containment within IVGLSs

**Figure 2:**
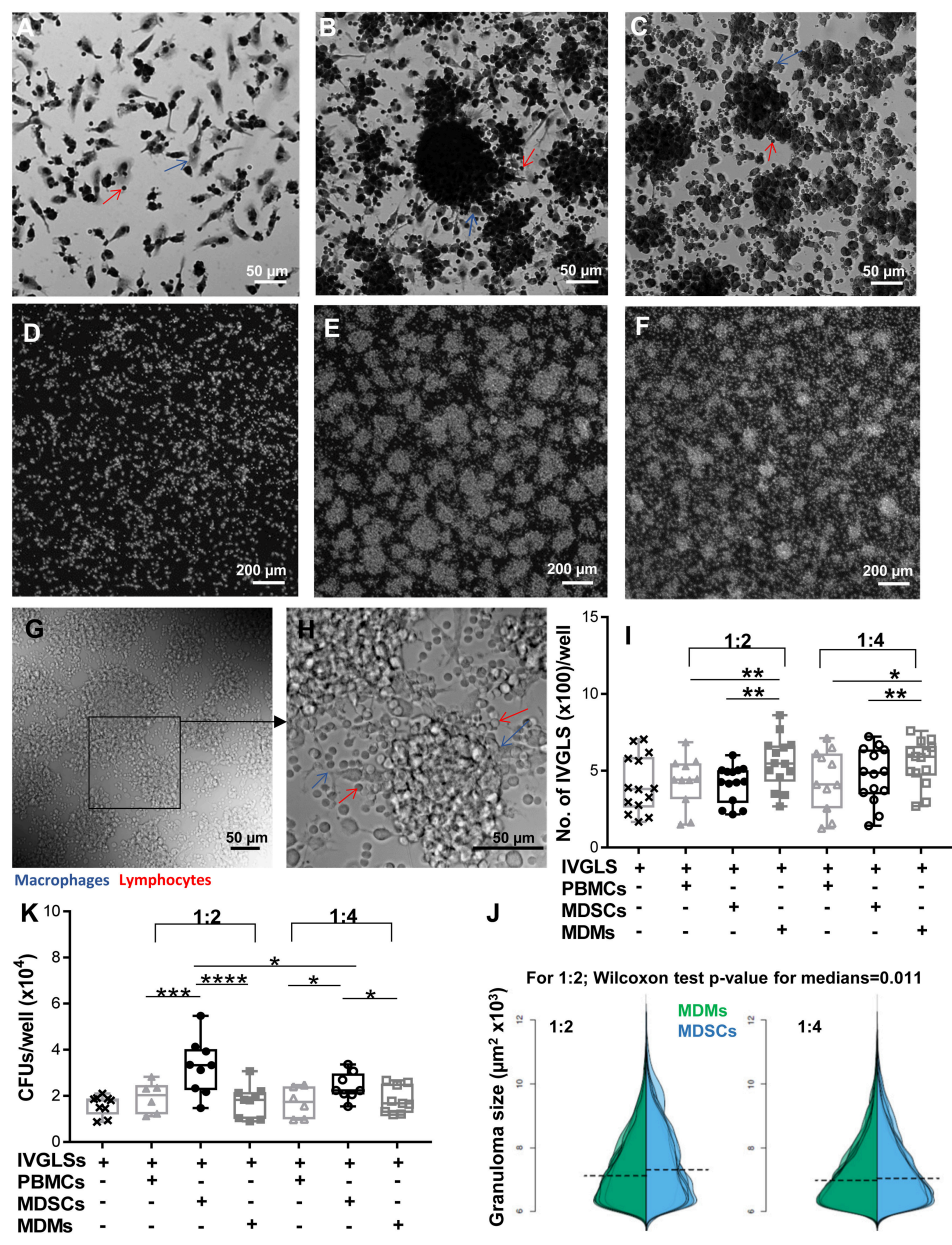
MDSCs alter the structure and bacterial containment of *in vitro* granuloma like structures (IVGLSs) induced by *Mtb* infection. Images captured using Arrayscan at day 7 of cultures after staining with Giemsa **(A–C)** or with the nuclear dye DAPI **(D–F)**. Images from uninfected wells **(A,D)** treated similarly to the *Mtb*-induced IVGLS wells **(B,C,E,F)**. Images represent IVGLS wells co-cultured for 48 h with MDSCs **(B,E)** or with MDMs **(C,F)**. **(G,H)** Bright field images of day 7 IVGLSs acquired with a confocal microscope. Z-stacked image of 30 focal planes **(H)**. Number and size of IVGLSs **(I,J)** and bacterial colony forming units (CFUs) **(K)** in IVGLSs at day 7 post generation and after co-culturing for the last 48 h with indicated cell subsets at distinct ratios. Added cells were at two ratios; 1:2 and 1:4 (added cells:number of PBMCs seeded for generation of IVGLSs). Each symbol corresponds to a single donor, shown are median±IQR. Data are from 5 independent experiments (*n* = 11–14 individuals) (representative for **A–H** and pooled for **I,J**) and from 3 independent experiments (pooled, *n* = 8 individuals) **(K)**; paired Student's *t*-test if not otherwise mentioned in figure, ^*^p ≤ 0.05, ^**^p ≤ 0.01, ^***^p ≤ 0.001, ^****^p ≤ 0.0001.

With the knowledge of reports specifying important roles of MDSCs at the site of infection (17, 20, 21), we wanted to delineate the outcomes of MDSC suppression in a disease-relevant setup. Effects of MDSCs on stability of *in vitro* generated granuloma-like structures and bacterial containment within these structures were ascertained. MDMs were used for comparative analysis because they usually harbor *Mtb* and tissue recruited inflammatory monocytes/macrophages share ontogeny with MDSCs (41). We employed an *in vitro* model developed by Agrawal et al. (35) and observed appearance of IVGLSs starting day 5 post generation (Supplementary Figures 5A–D). Within such structures, *Mtb* replicated upon extended incubation, as indicated by increased bacterial loads at day 7 post induction (Supplementary Figure 5E). This is in line with observations in other IVGLSs 42. We selected day 5 IVGLSs to initiate co-cultures with MDSCs/MDMs and observed responses 48 h afterwards, which was day 7 post IVGLS induction. IVGLSs became larger, yet less numerous, upon addition of MDSCs as compared to MDMs (Figures 2A–J, Supplementary Figure 5F). Addition of MDSCs to IVGLSs resulted in higher CFUs in IVGLSs, compared to MDM co-cultures (Figure 2K). This indicates that MDSCs facilitate *Mtb* growth or restrict anti-bacterial mechanisms within IVGLS either by providing a replication niche or by altering the microenvironment of the granulomas. Similarly, hsMDSCs induced higher bacillary loads in IVGLSs, albeit their propensity to enlarge these multicellular structures was inconclusive because of heightened variability among donors (Supplementary Figures 4B,C). Although majority of added cells remained viable upon extended co-culture with IVGLSs and apoptosis rates were similar between the two cell types, frequencies of necrotic MDSCs exceeded those of MDMs after 48 h of IVGLS co-cultures (Supplementary Figure 6). The effects on granuloma size and bacterial replication were more pronounced upon addition of higher numbers of MDSCs in all assays that is when ratio of MDSCs (added number):IVGLS (day 0 PBMCs number) was 1:2. In murine pulmonary TB models frequency of MDSCs at the site of infection parallels susceptibility to disease (17) and in humans their number decreases upon therapy (23). This was replicated in the IVGLS model suggesting that higher MDSC numbers may correlate with TB pathology in humans, possibly by tailoring *in situ* immune responses.

### IL-10 neutralization reduces MDSCs-induced *Mtb* growth within IVGLSs

**Figure 3:**
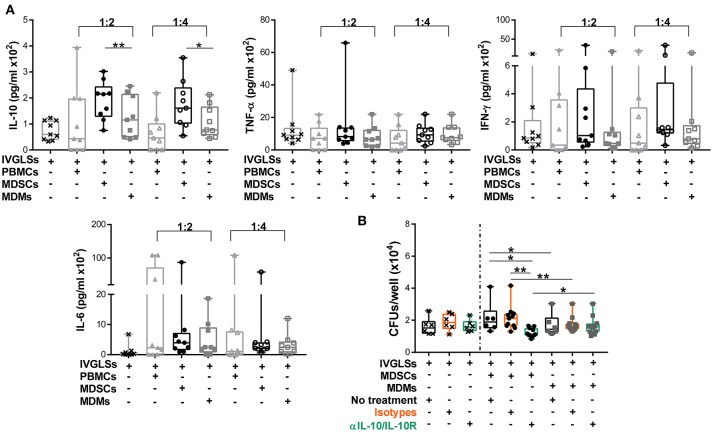
IL-10 promotes MDSC-driven bacterial replication in IVGLSs. **(A)** Cytokines from cell-free supernatants of IVGLSs at 48 h of co-cultures. **(B)** Bacterial burden at day 7 IVGLSs and after co-culturing with MDSCs/MDMs for the last 48 h and simultaneous IL-10 neutralization, all antibodies were used at 10 μg/ml. IVGLSs and IVGLSs co-cultured with MDSCs/MDMs contain different total cell numbers, hence data are separated by dotted line. Each symbol corresponds to a single donor, shown are median±IQR. Data are pooled from 3 independent experiments (*n* = 9 individuals) **(A)** and 4 independent experiments (*n* = 11 individuals) **(B)**; paired Student's *t*-test, ^*^p ≤ 0.05, ^**^p ≤ 0.01.

Stability of TB granulomas is positively regulated by TNF-α while IFN-γ is rather associated with increased protection to TB (2, 43). The anti-inflammatory cytokine IL-10 impairs *Mtb* specific immunity and promotes bacterial growth. The major sources of this cytokine are not well defined, specifically within granulomas (2, 44, 45). Modeling studies have suggested important roles of IL-10 in granuloma progression *via* macrophage polarization (46). We hypothesized that abundances of these cytokines could be altered by MDSCs and that in turn affected bacillary replication. Compared to MDMs, IL-10 levels in IVGLS co-cultures were indeed higher upon addition of MDSCs (Figure 3A). Concentration of IL-6 remained unchanged, although it is known to be released by MDSCs (17) and abundances of the pro-inflammatory cytokines TNF-α and IFN-γ, were also not significantly different in IVGLSs supplemented with MDSCs compared to MDMs. The amounts of TNF-α and IFN-γ positively correlate with IVGLSs size (35), however these cytokines were not altered by any of the cells added to IVGLSs in our study. To investigate whether IL-10 was critical for *Mtb* replication within IVGLSs co-cultured with MDSCs, we employed neutralizing antibodies. Combination of antibodies against IL-10 and IL-10 receptor A reduced bacterial growth compared with cultures receiving the isotype controls, specifically in MDSC treated IVGLSs (Figure 3B). We assume that an environment characterized by abundant IL-10 partially explains the mycobacteria promoting activity of MDSCs.

### MDSCs secrete differential amounts of inflammatory and regulatory cytokines/chemokines compared to MDMs after *Mtb* infection

**Figure 4:**
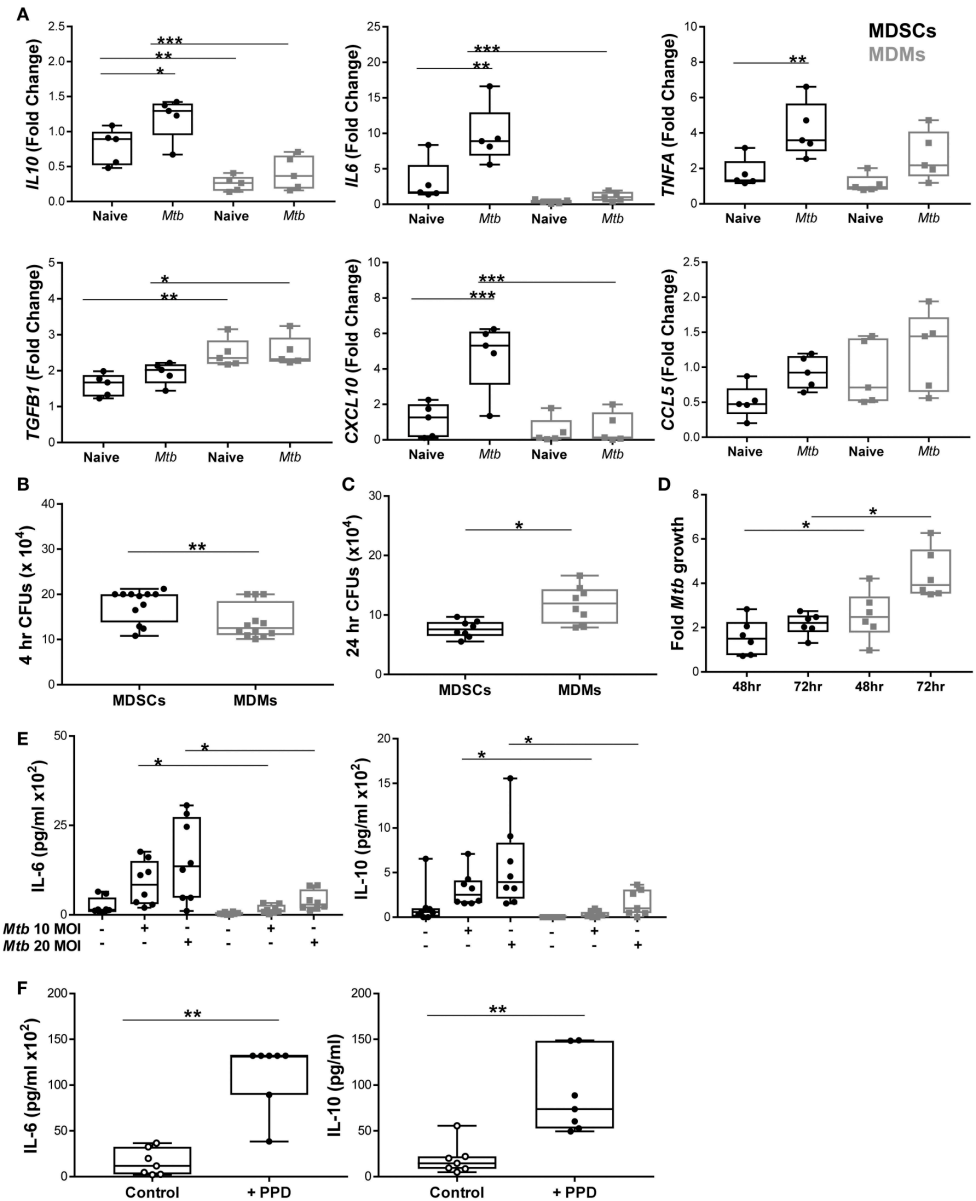
MDSCs secrete more abundant inflammatory and regulatory cytokines compared to MDMs upon *Mtb* infection. **(A)** Transcript abundances for cytokines and chemokines in naïve and at 4 h post *Mtb* infection (MOI 20) in MDSCs and MDMs were calculated for each donor compared to pooled data of all individuals using the Fluidigm platform (presented as fold change values). **(B)** Phagocytosis of *Mtb* (MOI 20) at 4 h post infection was assessed by plating CFUs for both cell types. **(C)** Bacillary loads at 24 h post *Mtb* infection (MOI 10). **(D)** Fold growth of *Mtb* (MOI 10) was calculated for 48 and 72 h by dividing CFU counts by respective 4 h counts. **(E)** IL-6 and IL-10 were measured from cell-free 24 h supernatants of *Mtb*-infected (MOI 10 and MOI 20) MDSCs and MDMs by ELISA. **(F)** IL-6 and IL-10 were measured from PPD stimulated MDSCs isolated from TB patients. Each symbol corresponds to a single donor, shown are median±IQR. Data are pooled from 2 independent experiment (*n* = 5 individuals) **(A)**, 4 independent experiments (*n* = 12 individuals) **(B)**, 3 independent experiments (*n* = 8 individuals) **(C)**, 2 independent experiments (*n* = 6 individuals) **(D)**, 3 independent experiments (*n* = 9 individuals) **(E)** and 6 independent experiments (*n* = 7 individuals) **(F)**; two-way ANOVA with Bonferroni correction **(A)** and paired Student's *t*-test **(B–F)**, ^*^p ≤ 0.05, ^**^p ≤ 0.01, ^***^p ≤ 0.001.

Murine MDSCs release both inflammatory and regulatory cytokines upon *Mtb* challenge, which prompted us to investigate how differential the source of IL-10, as observed in IVGLSs, was in human MDSCs. We profiled various immune relevant cytokines in MDMs and MDSCs infected with mycobacteria. mRNA expression of *IL10* and *IL6* was higher in infected MDSCs than in MDMs (Figure 4A), whereas transcripts for *TNFA* were comparable in the two cell types. We measured the expression level of TGF-β in addition to IL-10 as these cytokines are known to be expressed synergistically (47). Surprisingly, the expression of *TGFB* was lower in MDSCs. We monitored transcription of chemokines as these regulate recruitment of immune cells to sites of infection and could impact on dynamics of cells within granulomas. The chemokine CXCL-10 has been singled out as a powerful biomarker to distinguish active and latent TB from unexposed individuals (48). Expression of *CXCL10* was higher in infected MDSCs compared to MDMs. Levels of *CCL5* transcripts were comparable in the cells under investigation. This led us to investigate the phagocytic capacity of the two cell types. Bacillary burdens were higher in MDSCs after 4 h of H37Rv infection (Figure 4B). Interestingly, the higher bacterial load in MDSCs did not result in increased *Mtb* replication over time (observed at 24, 48, and 72 h) (Figures 4C,D). The lower replication of *Mtb* in MDSC monocultures and the opposite findings in case of IVGLSs (Figures 2K, 4D, respectively), suggests that MDSCs regulate *Mtb* replication within IVGLSs primarily through interactions with other immune cell types. Heightened transcription for *IL6* and *IL10* in MDSCs despite of lower bacterial replication resulted in more abundant cytokine concentrations in supernatants after 24 h of infection (Figure 4E). Thus, MDSCs have the ability to shift the balance of soluble mediators at the site of infection toward suppressive and regulatory cytokines, by releasing high amounts of IL-10 and IL-6. BCG resulted in similar patterns of IL-6 and IL-10 release, which further suggests that conserved pattern recognition receptors rather than unique virulence factors affected cytokine abundances in mycobacteria-infected MDSCs (Supplementary Figure 7). Further supporting this assumption, MDSCs isolated from TB patients secreted IL-6 and IL-10 after PPD stimulation (Figure 4F). The data emphasizes that MDSCs purified from human TB patients are similar with *in vitro* generated MDSCs with regard to their cytokine patterns induced by mycobacterial stimulation.

### MAP kinase and PI3K pathways play distinctive roles in cytokine secretion in MDSCs and MDMs with MAPK controlling mycobacterial replication in IVGLSs-MDSCs co-cultures

**Figure 5:**
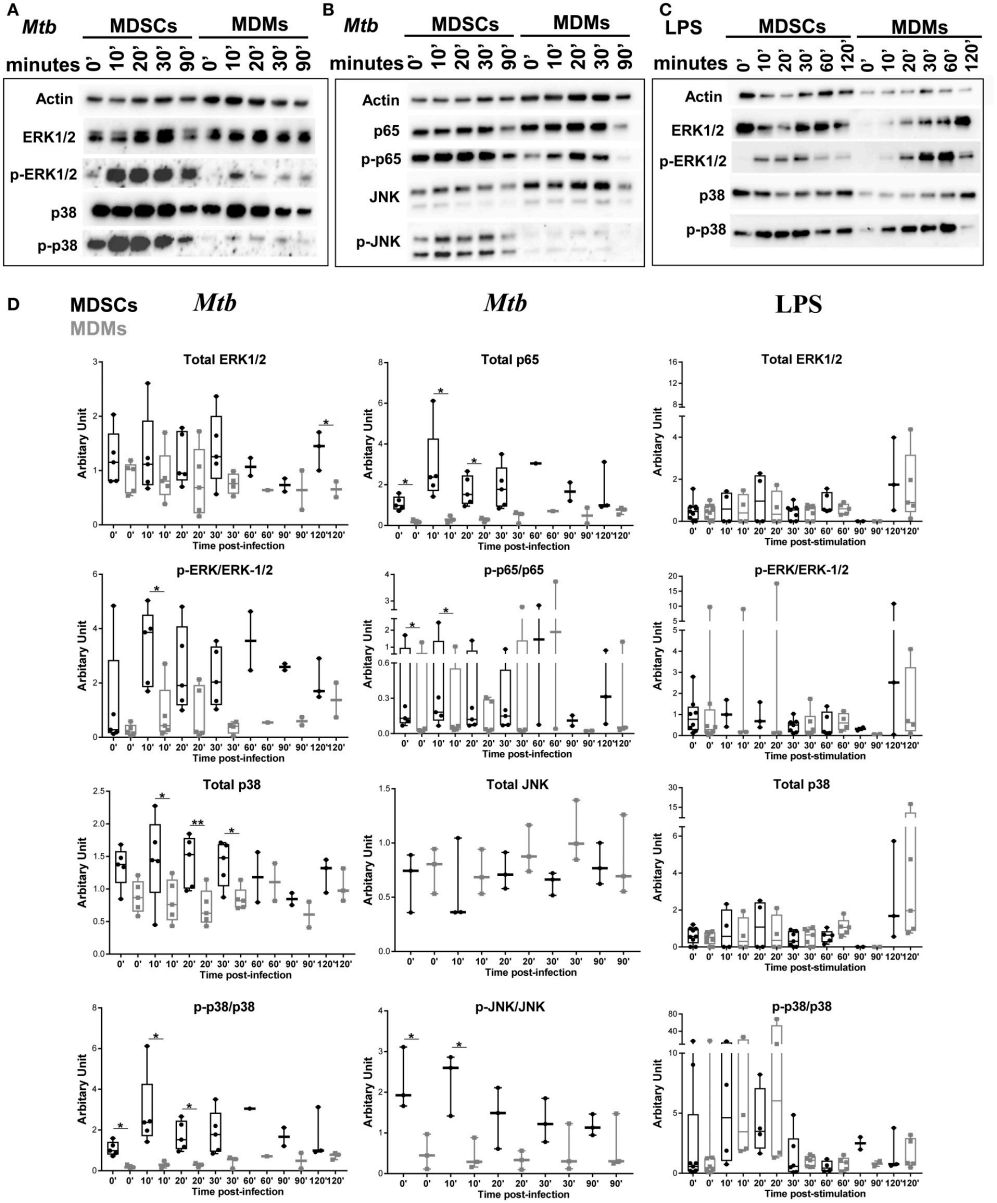
*Mtb* preferentially activates MAPK and NF-κB in MDSCs. Protein levels of p38, ERK1/2, JNK, and NF-κB and their phosphorylated counterparts at various time points post *Mtb* infection (MOI 20) **(A,B)** or LPS stimulation (25 ng/ml) **(C)**. Densitometry of the western blots was performed using ImageJ, values are plotted and normalized using actin. Data are representative of 2 independent experiments (*n* = 3–5 individuals) (**A, B**, *Mtb* **D**), 3 independent experiments (*n* = 3–7 individuals) (**C**, LPS **D**). Each symbol corresponds to a single donor, shown are median ± IQR, paired Student's *t*-test, ^*^p ≤ 0.05, ^**^p ≤ 0.01.

We further investigated the signaling pathways that contribute to differential cytokine release in MDSCs upon *Mtb* infection and focused on IL-6 and IL-10. Mycobacteria are recognized by various pattern recognition receptors (PRRs), including TLRs (TLR-2, TLR-4 and TLR-9) and C-type lectin receptors, to name a few (49). These events activate distinct mitogen-activated protein kinases (MAPK) and nuclear factor kappa B (NF-κB) for the generation of cytokines (50, 51). *Mtb* infection induced significantly higher phosphorylation of p38, ERK1/2, JNK, and p65 in MDSCs compared to MDMs, demonstrating that activation of MAPKs and NF-κB pathways was elevated in MDSCs (Figures 5A,B,D). Comparable results were achieved irrespective of the FCS starvation prior to infection (data not included). LPS, which is a bona-fide TLR-4 agonist, resulted in comparable activation of ERK and p38 pathways in MDSCs and MDMs (Figures 5C,D), therefore indicating that differences in MAPKs activation were specific for mycobacterial infection. Higher activation of MDSCs after mycobacterial infection might be a coordinated event with their high phagocytic capacity; however, early divergence in the magnitude and specificity of the signaling events rather suggest cell specific differences.

**Figure 6:**
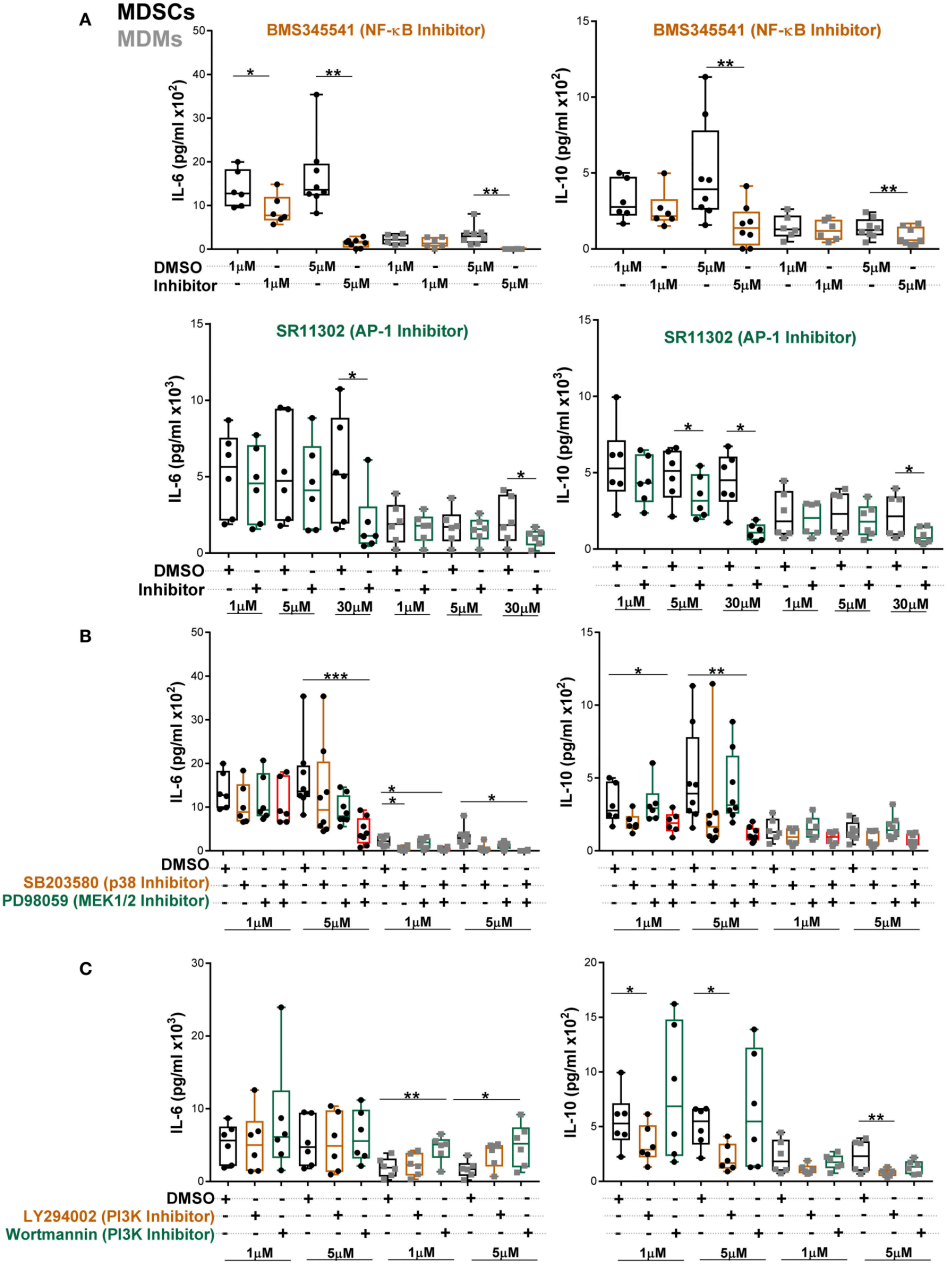
Abundant IL-6 and IL-10 release by MDSCs upon *Mtb* infection are regulated by NF-κB and MAPK pathways. IL-6 and IL-10 were measured in cell-free culture supernatants from *Mtb*-infected cells (MOI 10) by ELISA **(A–C)**. Cells were treated with indicated inhibitors and supernatants were collected at 24 h post infection. Each symbol corresponds to a single donor, shown are median ± IQR. Data are pooled from 3 independent experiments (*n* = 6–8 individuals); one-way ANOVA with Dunn's multiple correction or Wilcoxon test (if only 2 conditions were available), ^*^p ≤ 0.05 and ^**^p ≤ 0.01, ^***^p ≤ 0.001.

To further establish which of these pathways specifically contributed to heightened IL-6 and IL-10 secretion, MDSCs/MDMs were pre-treated with various inhibitors and cytokine levels were measured at 24 h post *Mtb* infection (Figures 6A–C). At low concentration of NF-κB inhibitor, IL-6 production in MDSCs was significantly inhibited (Figure 6A). However, IL-6 production in MDMs was only reduced by high concentration of inhibitors. The AP-1 inhibitor SR11302 reduced IL-6 secretion from both cell types (Figure 6A). Similar inhibition activity for IL-6 was displayed by another AP-1 inhibitor, Tanshinone IIa (Supplementary Figure 8). We observed that IL-10 secretion was furthermore regulated in MDSCs and MDMs by the afore-mentioned factors. These data suggest that both NF-κB and AP-1 contributes to IL-6 and IL-10 production in MDSCs.

To further delineate which MAPKs are involved, we employed specific ERK1/2 and p38 inhibitors. We found that single p38 or ERK1/2 inhibition did not alter IL-6 production in MDSCs (Figure 6B). However, the combination of p38 and MEK1/2 inhibitors significantly reduced IL-6, demonstrating that p38 and ERK1/2 mutually regulate IL-6 production in MDSCs. In MDMs single p38 inhibition reduced IL-6 release. IL-10 production from MDSCs was primarily inhibited by combination of p38 and MEK1/2 inhibitors. Of note, the differential outcomes of selective pathway inhibitors were associated with variable activation of corresponding signaling molecules in the two cell types and not imprinted by distinct cellular viability. There was no significant induction of cell death in either of cell types by various inhibitors at 1 and 5 μM concentrations (Supplementary Figure 9). We further observed significant increase in IL-6 secretion upon inhibition of the PI3K pathway by Wortmannin in MDMs (Figure 6C). However, there was no effect of PI3K inhibition on IL-6 production by MDSCs. PI3K inhibition by LY294002 reduced IL-10 secretion from both cell types, though more significantly from MDSCs. This may reflect contribution of the PI3K through GSK3-AP-1 (52) or via activation of ERK (53, 54) for IL-10 production after *Mtb* infection.

**Figure 7:**
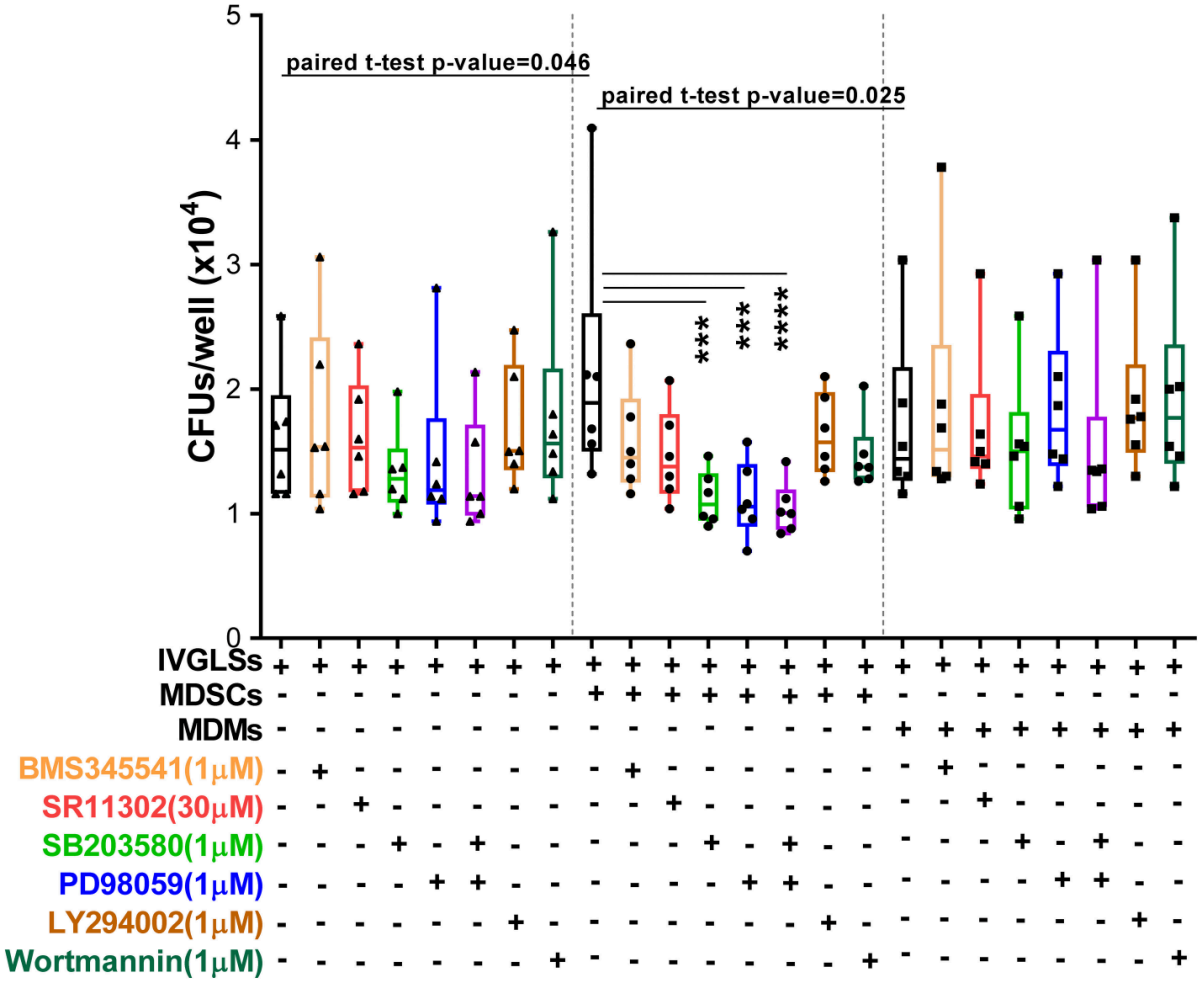
Interference with MAPK restricts bacterial replication in MDSCs co-cultured IVGLSs. Selected concentrations of inhibitors were added to co-cultures of IVGLSs and MDSCs/MDMs at the time of addition of the myeloid cells, that is day 5 post IVGLSs generation. CFUs were estimated after 48 h of co-culturing. Each symbol corresponds to a single donor, shown are median±IQR. Data are pooled from 2 independent experiment (*n* = 6 individuals); one-way ANOVA with Dunn's multiple correction [for conditions of (i) IVGLSs, (ii) MDSC co-cultures, and (iii) MDM co-cultures], ^***^p ≤ 0.001, ^****^p ≤ 0.0001. Paired Student's *t*-test was performed only for untreated conditions.

Inhibitors of p38 and MEK1/2 applied at 1 μM concentrations reduced *Mtb* growth specifically in MDSCs-IVGLSs co-cultures (Figure 7), thereby indicating that these signaling pathways likely control the IL-10 release and the subsequent *Mtb* growth. Taken together, our data indicate that MDSCs employ both p38 and ERK1/2 and hence activate AP-1/NF-kB for IL-6 and IL-10 production with impact on bacterial containment within granuloma-like structures.

### MDSCs upregulate PD-L1 to regulate CD3^+^ lymphocyte proliferation and leave *Mtb* growth unchanged

**Figure 8:**
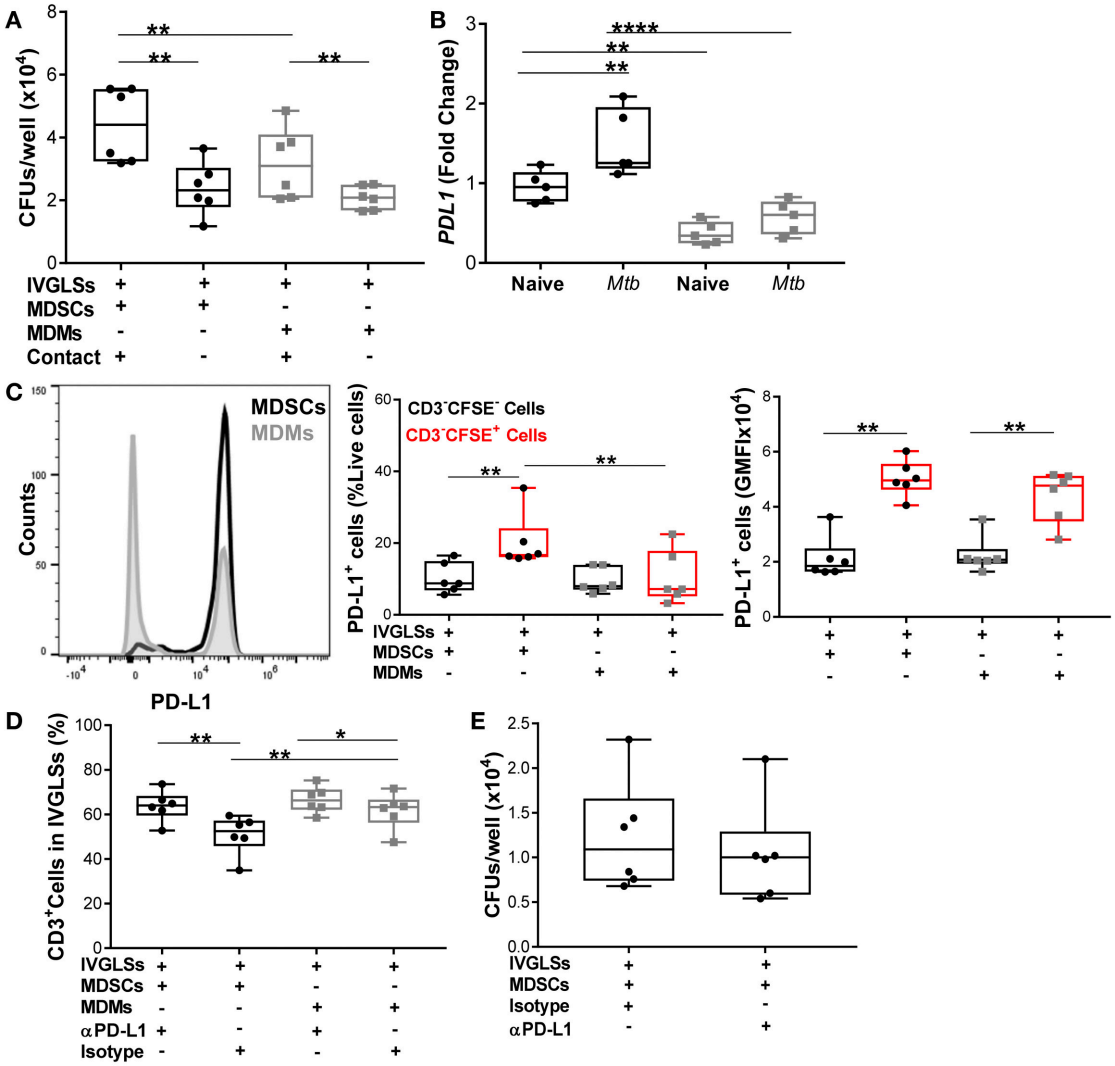
Numbers of CD3^+^ cells but not *Mtb* growth are regulated via PD-L1 in IVGLSs-MDSCs co-cultures. **(A)** Bacterial loads in IVGLSs at 48 h following addition of MDSCs/MDMs with or without direct contact (using trans wells), that is at day 7 post IVGLSs generation. **(B)** *PDL1* expression in monocultures of MDSCs and MDMs at 4 h post *Mtb* infection (MOI 20). **(C)** CFSE-tracked MDSCs or MDMs were co-cultured with IVGLS and PD-L1 surface expression was monitored in CFSE^−^ and CFSE^+^ fractions of cells from each culture condition, where CFSE^−^ cells represent myeloid cells of IVGLSs present before initiation of the co-cultures. **(D)** Frequency of CD3^+^ T-cells at day 7 IVGLSs, that is after 48 h IVGLS post co-culturing with MDMs/MDSCs, with or without simultaneous PD-L1 neutralization (10 μg/ml αPD-L1 or isotype control). **(E)** Day 7 bacterial loads in IVGLSs wells and applying for the last 48 h PD-L1 neutralization and MDSC co-culturing. Data are pooled from 2 independent experiment (*n* = 5 individuals) **(B)** and (*n* = 6 individuals) **(A,C–E)**, shown are median±IQR; two-way ANOVA with Bonferroni correction **(B)** and paired Student's *t*-test **(A,C–E)**, ^*^p ≤ 0.05, ^**^p ≤ 0.01, ^****^p ≤ 0.0001.

Neutralization of IL-10 reduced bacillary growth within IVGLSs, however we also observed that MDSCs and MDMs both required direct T-cell contact to promote bacterial growth (Figure 8A). We quantified levels of PD-L1, which is known to be upregulated on tumor-infiltrating MDSCs (55, 56) and contributes to exhaustion of T-cell immunity by cell-cell contact (57). *PDL1* transcripts were more abundant in steady state in MDSCs compared to MDMs and the *Mtb* infection further upregulated expression of this gene in MDSCs (Figure 8B). We determined the PD-L1 surface expression on cells purified from co-cultures of MDSCs/MDMs with IVGLSs. Although the surface expression (gMFI) of PD-L1 was similar for MDSCs and MDMs (CFSE-labeled), the frequencies of PD-L1 expressing cells were higher in case of IVGLSs supplemented with MDSCs compared with cultures receiving MDMs (Figure 8C), suggesting a possible contribution of this co-regulatory molecule to immune suppression. To investigate the outcome of this regulation on T-cell responses and *Mtb* growth, neutralizing antibodies against PD-L1 were employed. This treatment resulted in more abundant CD3^+^ lymphocytes, however the overall growth of *Mtb* within IVGLSs remained unchanged (Figures 8D,E). Thus, *Mtb* growth in IVGLSs is regulated via contact-dependent mechanisms and MDSCs employ co-inhibitory receptors to regulate lymphocyte survival and/or replication, but neutralization of PD-L1 alone is insufficient to restrict bacterial growth. It has been reported that PD-L1 blockade may not reduce the suppressive activity of MDSCs (58), therefore it remains to be established which specific contact-dependent interactions of MDSCs with cells within granulomas are decisive for mycobacterial survival.

## Discussion

Using *in vitro* differentiated MDSCs we demonstrated that this monocytic subset readily phagocytosed *Mtb*, albeit allowing reduced bacillary replication in monocultures, abundantly released IL-10 and employed this cytokine for promoting growth of *Mtb* within IVGLSs. MDSCs have been recently described in TB (13–15, 17–23); however particularly in humans outcome of their direct interactions with *Mtb* as well as their roles at the site of pathology remain enigmatic. Our study unveils responses of MDSCs to virulent mycobacterial infection and proposes roles of MDSCs in granuloma progression. Granulomas in TB function to contain *Mtb*, but may also facilitate bacterial replication within and drive disease progression upon caseation (8). We hypothesized that MDSCs alter granuloma stability. Using an *in vitro* model for human granulomas we observed that MDSCs, in contrast to MDMs, promoted expansion of such multicellular structures and *Mtb* replication. The sera employed for MDSC generation may however modulate their capacity to alter granuloma size as expansion of granuloma-like structures upon hsMDSCs addition was inconclusive. While *Mtb* preferentially replicates in MDMs, MDSCs affect growth of bacteria within granulomas via IL-10. Interestingly, *in vitro* granuloma-like structures generated from latent TB individuals develop faster, are larger and better in controlling mycobacterial replication (42). This appears counterintuitive in view of our findings as LTBI individuals present, albeit at very low frequencies, MDSCs in their blood (23). In LTBI patients potent antigen specific T-cell responses may overcome the effects of scantly detectable MDSCs. Alternatively, granuloma-like structures lack MDSCs under the experimental conditions applied by Guirado et al. (42). Considering our study design and the fact that blood donors included in analyses were *Mtb* naïve, based on regional epidemiological data, it is difficult to conclude on how scant MDSCs in LTBI individuals would affect IVGLSs size and bacillary containment. The current experimental setup mimicked recruitment of MDSCs to developing granulomas, as it could occur in active TB. Our observations highlight that MDSCs (i) could be regulators of granuloma biology, (ii) employ soluble factors and direct cell-to-cell contact to enable *Mtb* growth, (iii) specifically activate MAPK and NF-κB pathways in response to mycobacteria to regulate cytokine release, and (iv) poorly support intrinsic *Mtb* replication yet may contribute to bacillary persistence.

IFN-γ and TNF-α are associated with protective immunity, both in mice and humans and sufficient TNF-α is required for maintenance of granuloma structure and limits reactivation of latent TB. Both cytokines are secreted by CD4^+^ and CD8^+^ T-cells as well as by additional lymphocyte subsets, however deficiency of the CD4^+^ lymphocytes cannot be compensated for in TB (2). The ability of naïve and *Mtb*-infected MDSCs to suppress CD4^+^ and CD8^+^ lymphocyte proliferation, as observed upon polyclonal stimulation and co-culture, suggested that they could alter frequencies and products of these lymphocyte populations. Using IVGLSs, we could not detect any impact of the MDSCs on the overall abundances of IFN-γ and TNF-α. This may indicate that the MDSCs do not alter antigen specific cytokine release, or alternatively that sources of these cytokines inside *in vitro* generated granuloma-like structures were unresponsive to MDSCs. While we measured soluble TNF-α aiming to ascertain a possible role in IVGLS stability, a role for transmembrane TNF in human MDSCs, including suppression of lymphocytes, as demonstrated in mice (22), cannot be excluded. Irrespective of their suppressive mechanisms, human MDSCs did not modify abundances of TNF-α in IVGLSs. Suppression of T-cell proliferation through MDSC-derived reactive oxygen radicals may occur during mycobacterial infection. In mice, nitric oxide (NO) released by monocytic Ly-6G^−^Gr-1^+^ cells and IL-1R-dependent NO production by CD11b^+^Ly-6C^int^Ly-6G^−^ cells control lymphocyte proliferation (18, 19). NO is hardly measurable in mycobacteria-infected human macrophages in normoxic environment (59), however propensity of human MDSCs to produce NO and ROS and their functional relevance should be considered.

Addition of MDSCs to IVGLSs increased abundances of the immunosuppressive cytokine IL-10. This cytokine is present in lungs of TB patients (46), inhibits phagosome maturation in *Mtb*-infected macrophages (60), suppresses CD4^+^ T-cell proliferation (61) and initiates type I interferon responses (6). Depletion of IL-10 promotes resistance to TB in mice by inducing more infiltrating CD4^+^ T-cells in infected lungs (62). Many immune cells secrete IL-10, however the source of this cytokine in TB granulomas has not been clearly established. We showed that human MDSCs, including cells *ex vivo* purified from TB patients, abundantly produced this cytokine upon mycobacterial stimulation. Using *in vitro* generated MDSCs we demonstrated that IL-10 production is primarily controlled by selected MAPKs and NF-κB. Whether sera source used for MDSC differentiation impacts on the IL-10 release and the upstream pathways remains to be verified. A role for IFN-I in regulation of IL-10 in MDSCs cannot be excluded. We detected more abundant transcripts of *CXCL10*, a bona-fide gene regulated by IFN-I, in MDSCs. By releasing IL-10, MDSCs partly regulated *Mtb* growth within IVGLSs. An autocrine regulation is unlikely because *Mtb* replication within MDSC monocultures was reduced. Replication rates were much lower in MDSCs than those observed in MDMs despite the former showing a high capacity to phagocytize *Mtb*. Although MDSCs show less phagocytosis of and consecutively favor survival of other microbes, notably *Klebsiella penumoniae* (63), more investigations are necessary to identify survival mechanisms of *Mtb* inside MDSCs. MDSCs may affect responsiveness of other myeloid cells within IVGLSs, for example macrophages containing mycobacteria. Control of microbicidal activity of myeloid cells via MDSC-derived IL-10 has been reported recently (63). Alternatively, MDSCs can also decrease MHC-II expression of macrophages (64). Macrophages are also reported to enhance secretion of IL-10 by MDSCs and in return MDSCs down-regulate in a contact-dependent manner IL-12 production by macrophages in tumor models (65). Whether such mechanisms are operative in IVGLSs awaits clarification. *Mtb* is able to replicate extracellularly (66, 67) and thus inhibition of phagocytosis, as noted for *K. pneumoniae* (63), would again favor bacterial growth in presence of MDSCs. Of note, within IVGLSs MDSC undergo rather necrosis. Although we do not have evidence regarding infectivity of the necrotic MDSCs, cytolysis could precondition *Mtb* for accelerated replication (67, 68).

Human MDSC, like their murine counterparts (17), released abundant IL-6, albeit without significant impact on overall concentration in IVGLSs. This cytokine is elevated in advanced pulmonary TB patients (69), it positively correlates with MDSC frequencies in malignant cancer patients (70) and drives mobilization or expansion of MDSCs (71, 72). Therefore, high secretion of IL-6 by mycobacteria-infected MDSCs could represent a positive feedback for maintenance and recruitment of MDSCs at the site of infection. Antitumor T-cell response and IFN-γ secretion are both restored by administration of anti-IL-6 receptor antibody with subsequent down-regulation of MDSCs (73). Such a strategy to limit MDSC generation may be advisable in TB, since IL-6 inhibits detrimental IFN-I in TB (74) and induces IFN-γ during early mycobacterial infection (75). IL-6 specific contributions to TB granulomas, notably lesion stability, and outcome remain undefined.

Activation of MDSCs in various cancer models involves multiple signaling pathways, including RAF/MAPK, PI3K/AKT, JAK/STAT, and NF-κB (76). In sepsis, TLR4 independent and myeloid differentiation primary response protein 88 (MyD88) dependent activation controls MDSC functions (77). We observed faster, and more pronounced activation of several MAPKs and NF-κB pathways in *Mtb*-infected MDSCs compared to MDMs. Differences in signaling kinetic have not been detected for LPS, despite upregulation of CD14 on MDSCs in an inflammatory environment (78). While MDSCs indeed present more abundant signaling molecules at steady state, further studies should clarify the precise upstream receptors which sense mycobacterial cues and promptly switch on signaling in MDSCs. In mice MyD88-dependent recruitment of MDSCs and IL-1R-dependent iNOS activation affects T-cell immunity following BCG vaccination (18). Moreover, murine MDSCs produce IL-1 (17) and could in autocrine or paracrine way affect signaling activity. Expression and involvement of the MyD88/IL-1R in human MDSC responses to mycobacteria awaits clarification. In our study, MAPKs and NF-κB pathways cumulatively drive secretion of IL6 and IL-10 after *Mtb* infection. We observed that MDSCs are more sensitive to NF-κB mediated IL-6 inhibition and coordinated inhibition of p38 and MEK1/2 is required to inhibit secretion of IL-6 and IL-10 in MDSCs. PI3K inhibition differentially upregulated IL-6 secretion by MDMs but not by MDSCs, whereas IL-10 secretion by MDSCs was reduced significantly. This suggests that the two cell types exhibit preferential signaling for targeted cytokine release upon mycobacterial sensing. Further studies, including identification of subcellular localization of *Mtb* in MDSCs, are required to delineate precise mechanisms of MDSC activation in TB.

Therapies targeting the PD-1/PD-L1 pathway, or checkpoint blockade inhibitors, represent a breakthrough in immunotherapy and improve clinical outcomes in certain cancers substantially (79). This strategy has been extended to chronic infectious diseases. Checkpoint inhibitors improve HIV-1 specific immunity in a subset of patients and promote clearance of hepatitis B virus in murine models of persistent infection (80, 81). MDSCs express PD-L1 and its blockade restores T-cell activation mediated by tumor infiltrating MDSCs with down regulation of IL-6 and IL-10 (82). However, other studies have failed to validate PD-L1-mediated suppression by MDSCs (58, 83, 84). We found that *PDL1* is upregulated in *Mtb*-infected MDSCs and when IVGLSs are co-cultured with MDSCs, more PD-L1 positive cells are present in the system. Checkpoint blockade therapy of IVGLSs co-cultured with MDSCs resulted in more abundant CD3^+^ T-cells, suggesting increased lymphocyte viability and/or proliferation in absence of the PD-1/PD-L1 co-inhibitory axis. Yet, PD-L1 neutralization left mycobacterial growth unchanged, despite higher lymphocyte frequencies. The relevance of PD-1 for T-cell control of TB has been demonstrated in murine model (85) and recent studies in active TB patients suggested that the antagonists to PD-1 rescue suppressive activity of MDSCs, primarily by interfering with the PD-1/PD-L2 cross-talk (86). These investigations also show that IFN-γ restricts suppressive capacities of human MDSCs if the cytokine is available during generation of MDSCs. Although in our system MDSCs exhibited potent lymphocyte suppression post-generation, it cannot be excluded that the presence of IFN-γ in IVGLSs may limit suppression via PD-1. Possibly other cell-cell contact mechanisms, independent of PD-1 impact the bacillary growth in presence of MDSCs. The cytolytic capacities of lymphocytes deserve further investigations. Moreover, MDSCs expand regulatory T-cells (87) and these may also affect anti-mycobacterial defense.

Our results offer novel views on cellular diversity and functions of myeloid cell subsets in granulomas. The granuloma microenvironment in TB patients contains factors which could reprogram recruited monocytes into MDSCs, notably cytokines such as GM-CSF and IL-6 (84), as well as lipid mediators, including PGE_2_ (88, 89). In a pro-inflammatory environment, MDSC differentiation may also encompass licensing by GM-CSF and IFN-γ leading to stable expression of suppressive factors, as reported for CD14^+^ monocytes (90). These soluble mediators are present in TB lesions and may contribute to local MDSC genesis. Alternatively, MDSCs may be generated in the bone marrow in active TB, as demonstrated in murine models (20) and populate granulomas following their release into blood stream. *In situ* genesis and differentiation in bone marrow are also not mutually exclusive processes. Whether the presence of MDSCs in TB granulomas drives pathology or if these cells are a consequence of unresolved inflammation, subsequently exacerbating tissue damage remains to be established. Our data reveal that *in vitro* generated human MDSCs readily phagocytosed mycobacteria, yet they do not offer a niche for fast replication, as macrophages do. Effects of sera used for generation of MDSCs on *Mtb* permissiveness was not addressed in the current study. Moreover, these findings need validation with MDSCs isolated from the site of infection in TB patients. It is tempting to speculate that MDSCs have dual detrimental roles in TB, supporting bacterial persistence inside granulomas; via intrinsic mechanisms, which await clarification and bacterial replication; via paracrine mechanisms including release of IL-10 and cell to cell contact. Such scenarios may arise in other chronic infectious diseases as well. While we largely focused on monocytic suppressor cells, involvements of granulocytic MDSCs in TB may as well be critical for the disease outcome, including granuloma features. In murine TB both types of MDSCs exert suppressive activity and in humans particularly granulocytic MDSCs expand during active TB (14). Clarifying roles and outcomes of direct interactions between granulocytic MDSCs and *Mtb* warrant future investigation.

By promoting *Mtb* replication within IVGLSs, monocytic MDSCs qualify as potential targets for host directed therapies (HDT). Identification of the signaling pathways driving generation of regulatory cytokines further positions MDSCs as druggable candidate. Such therapies in TB are spearheaded by ongoing work focused on MDSCs in cancer (91). Various drug classes could be tested in fast-tracked clinical trials and eventually be repurposed for HDT in TB.

## Ethics statement

The study performed by utilizing samples from healthy donors was approved by the Ethics Committee of Charitè University Hospital (Charitè Universitätsmedizin) Berlin; approval number EA2/083/14. Donors were kept anonymous. The study involving TB patient samples was approved by Human Research Ethics Committee of Stellenbosch University; approval number S16/10/212. All sensitive patient information was kept confidential to clinical research personnel only. The study was performed in accordance with the Helsinki Declaration.

## Author contributions

The study was conceived by AD. NA and IS designed, conducted, and analyzed experiments with support from AD, NdP, GW, MI and SK. GP, LL and LK provided support with investigations of the signaling pathways, Fluidigm analysis and patient data respectively. SB provided support with selected BSL3 experiments. JW supported statistical analysis. NA and AD wrote the manuscript with valuable comments from IS, GP, LL, NdP, MI, JW, and SK.

### Conflict of interest statement

The authors declare that the research was conducted in the absence of any commercial or financial relationships that could be construed as a potential conflict of interest.
