# Supplementary material for: Human Monocytic Suppressive Cells Promote Replication of Mycobacterium tuberculosis and Alter Stability of in vitro Generated Granulomas
Source: Front Immunol. 2018 Oct 23;9:2417. doi: 10.3389/fimmu.2018.02417 (PMC6205994; doi:10.3389/fimmu.2018.02417)
Supplement: Supplementary file 1 [file Data_Sheet_1.docx]

**Supplementary Information**

**Human monocytic suppressive cells promote replication of *Mycobacterium tuberculosis* and alter stability of *in vitro* generated granulomas**

**Neha Agrawal^1#^, Ioana Streata^2#^, Gang Pei^1^, January Weiner^1^, Leigh Kotze^3^, Silke Bandermann^1^, Laura Lozza^1^, Gerhard Walzl^3^, Nelita du Plessis^3^, Mihai Ioana^2^, Stefan H.E. Kaufmann^1^, Anca Dorhoi^1,4,5*^**

1. Max Planck Institute for Infection Biology, Department of Immunology, Berlin, Germany

2. University of Medicine and Pharmacy Craiova, Human Genomics Laboratory, 200638 Craiova, Romania

3. Division of Molecular Biology and Human Genetics, Department of Biomedical Sciences, Faculty of Medicine and Health Sciences, SAMRC Centre for Tuberculosis Research, DST and NRF Centre of Excellence for Biomedical TB Research, Stellenbosch University, Tygerberg, South Africa

4. Institute of Immunology, Federal Research Institute for Animal Health, Friedrich-Loeffler-Institut (FLI), Insel Riems, Germany

5. Faculty of Mathematics and Natural Sciences, University of Greifswald, Greifswald, Germany

# Equal contribution

*Correspondence:

Anca Dorhoi: anca.dorhoi@fli.de

**Supplementary Figure S1**


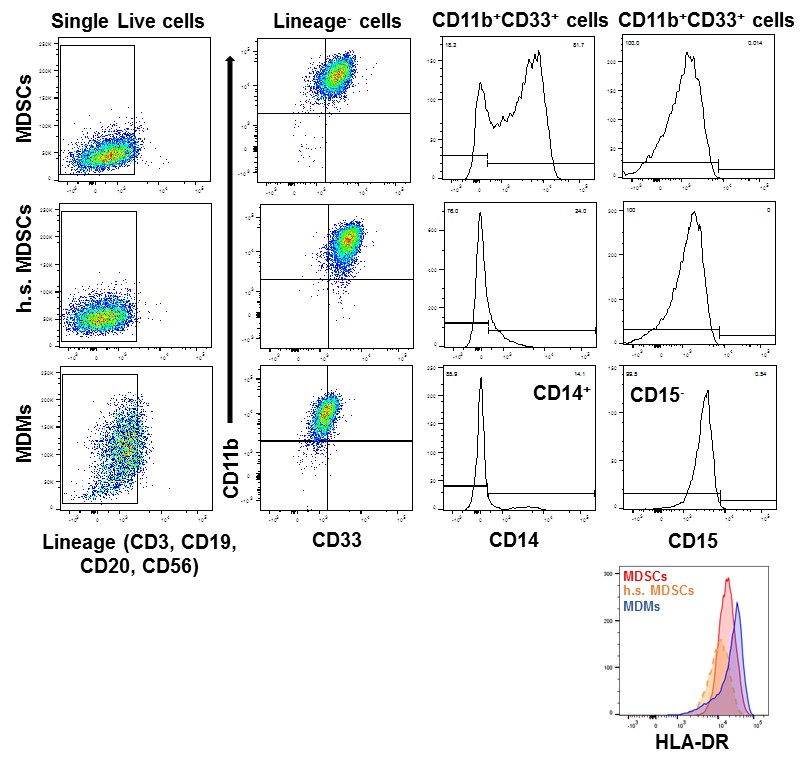


**Phenotype of *in vitro* generated cells with various serum sources.** The following cells were generated from the same donor: MDSCs, MDMs (both according to protocols detailed in the Materials and Methods section) and hsMDSCs (similar protocols as for MDSCs, fetal serum was replaced by human serum, hs) and analyzed by multi-parameter flow cytometry. Gating strategy as well as representative dot-plots and histograms are presented. Data is representative of 9 individuals.

**Supplementary Figure S2**


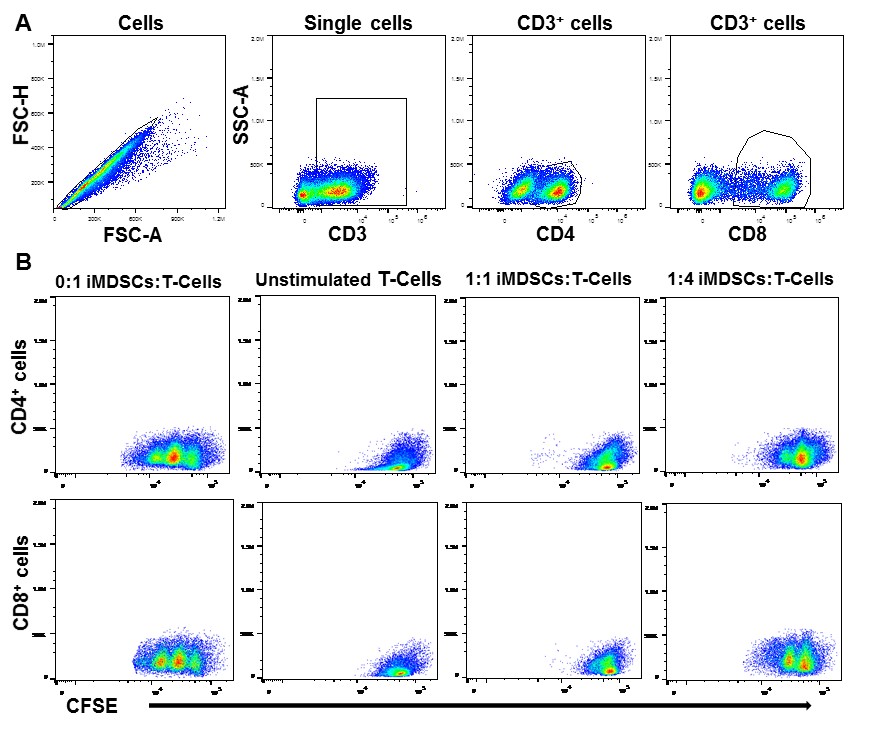


**Gating strategy for assessment of T-cell proliferation in the CFSE dilution assay.** Cells were processed according to the protocols detailed in the Materials and Methods section. (A) Gating strategy for detection of CD4^+^ and CD8^+^ lymphocytes co-cultured with MDSCs. (B) Representative dot-plots depicting CFSE staining patterns of the CD4^+^ and CD8^+^ lymphocytes co-cultured with various ratios of *Mtb*-infected MDSCs, polyclonally stimulated or left untreated. Data is representative of 6 individuals.

**Supplementary Figure S3**


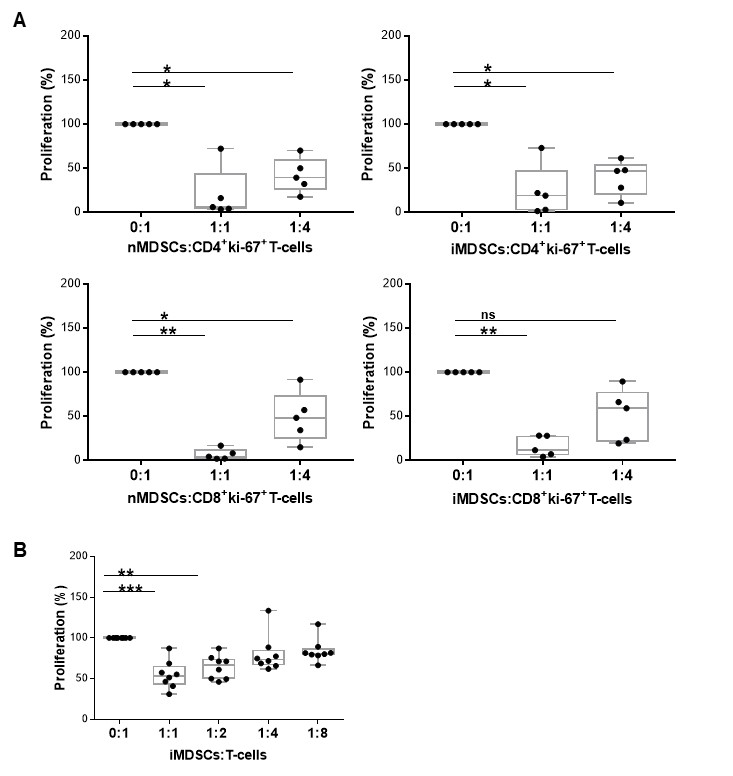


**Mycobacteria-infected MDSCs suppress T-cells.** (A) Ki-67^+^ staining of T-cells co-cultured with various ratios of *Mtb*-infected (MOI 10) MDSCs. (B) [^3^H]-Thymidine incorporation by T-cells co-cultured with various ratios of BCG-infected MDSCs. Each symbol corresponds to a single donor, shown are median±IQR. Data is pooled from 2 independent experiments (n=6 individuals) (A) and from 3 independent experiments (n=8 individuals) (B); paired Student’s t-test, *p≤0.05, **p≤0.01, ***p≤ 0.001.

**Supplementary Figure S4**

**
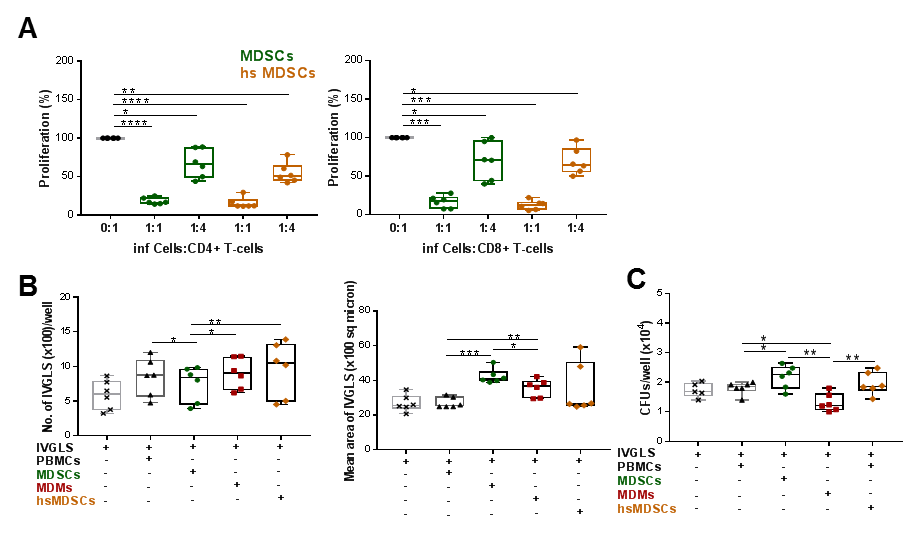
**

**MDSCs generated with human serum (hsMDSCs) recapitulate key features of MDSCs generated with FCS.** (A) Proliferation of polyclonally stimulated, CFSE-labeled T-cells co-cultured with *Mtb*-infected cells (MOI 10) for 72 hr. Number of T-cells was kept constant for all conditions with varying amount of MDSC/hsMDSCs addition; no cells (0:1), 1/4 cells (1:4) and equal cells (1:1). (B) IVGLSs co-cultured for 48 hr with PBMCs/MDSCs/MDMs/hsMDSCs (at 1:2 ratio) were quantified using high throughput microscopy (as described in Materials and Methods). (C) Bacterial loads in IVGLSs at 48hr post addition of indicated cell types at 1:2 ratio. Each symbol corresponds to a single donor, shown are median±IQR. Data is pooled from 2 independent experiments (n=6 individuals). Paired Student’s t-test, *p≤0.05, **p≤0.01, ***p≤ 0.001, ****p≤ 0.0001.

**Supplementary Figure S5**

**
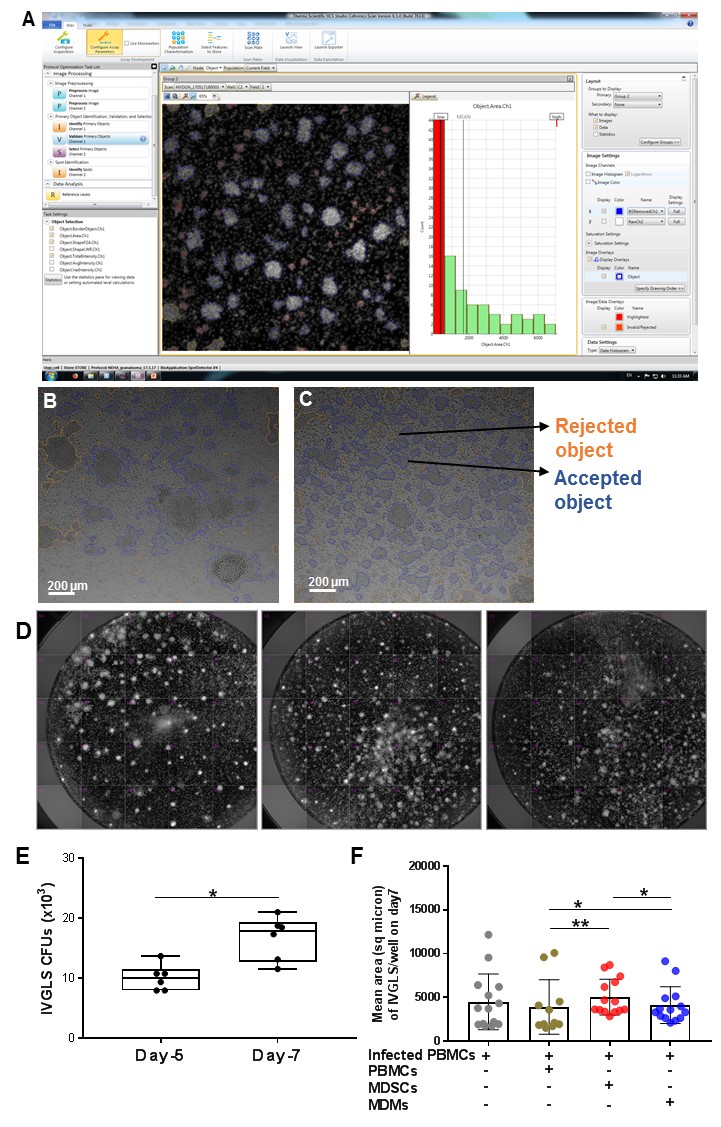
**

**Analysis and features of IVGLSs.** (A) Print screen of the HCS Studio software indicating the workflow and settings applied for the analysis of the IVGLSs. The nuclear staining (DAPI) was used to define regions of interest (ROIs)/field. Images were acquired on the Arrayscan platform at 5X magnification. For each sample a total of 24 fields were captured (24 fields/well, 48 well plate) at 5X magnification. Each donor was processed in triplicates. (B and C) Representative bright field images of IVGLSs from the same donor, samples were processed at 48 hr following addition of MDSCs (B) or MDMs (C) to IVGLSs, 5X magnification. (D) Representative composition images (individual fields assembled to obtain an overview of the whole well) of day 5 IVGLSs from 3 different donors, acquisition on the Arrayscan platform, 5X magnification. (E) Bacterial burdens in IVGLSs were estimated at day 5 and day 7-post generation (that is post infection of the PBMCs with *Mtb* at MOI 0.005). (F) Area of IVGLS co-cultured with MDSCs, MDMs or PBMC, MDMs/MDMs/PBMCs:IVGLSs ratio 1:2. Each symbol corresponds to a single donor, shown are median±IQR. Data is pooled from 2 experiments (E) (n=6 individuals) and from 5 experiments (F) (n=11-14 individuals); paired Student’s t-test, *p≤0.05, **p≤0.01.

**Supplementary Figure S6**

**
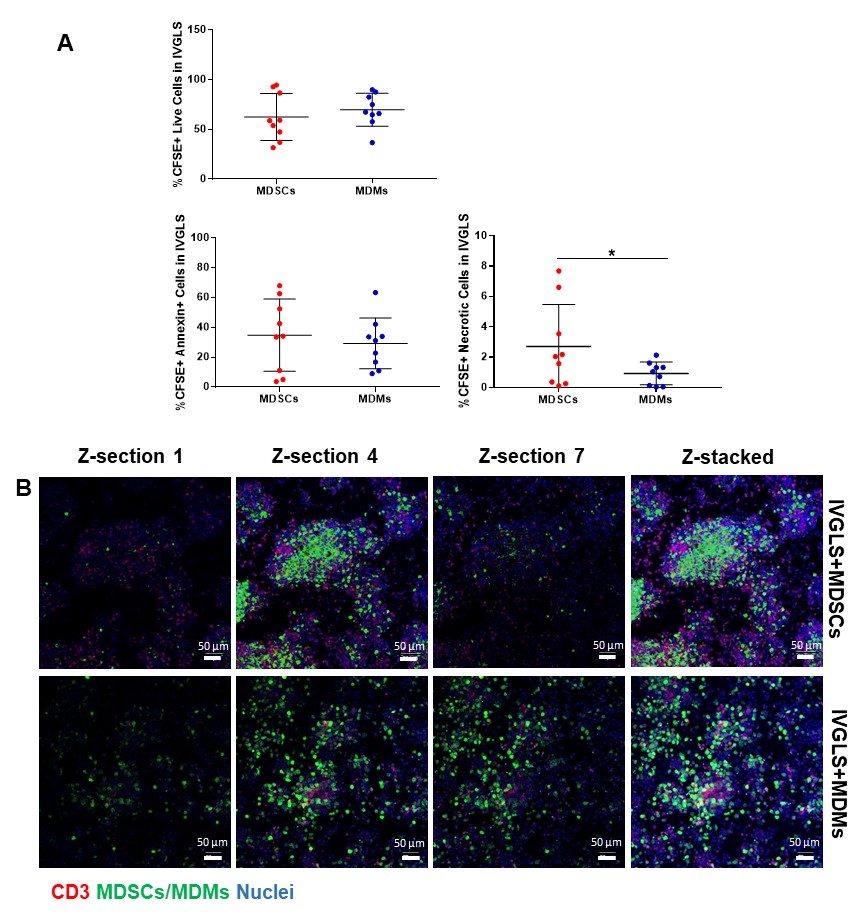
**

**Comparable** **viability of MDSCs and MDMs at 48 hr of co-culture with IVGLSs.** CFSE-labeled MDSC/MDMs were co-cultured with day 5 IVGLSs and 48 hr later cells were isolated and analyzed for viability. (A) Frequencies of apoptotic, necrotic and viable MDSCs/MDMs were evaluated by flow cytometry based on Annexin V and Live/Dead staining. (B) Z-sections and Z-stacked images of IVGLS co-cultured with MDSCs and MDMs (CFSE-tracked, green) and strained for T lymphocytes (CD3, red), nuclei in blue (DAPI). Images were acquired at 3 μm distance between consecutive focal planes, 15X magnification. Each symbol corresponds to a single donor, shown are median±IQR. Data is pooled from 3 experiments (n=9 individuals); paired Student’s t-test, *p≤0.05.

**Supplementary Figure S7**


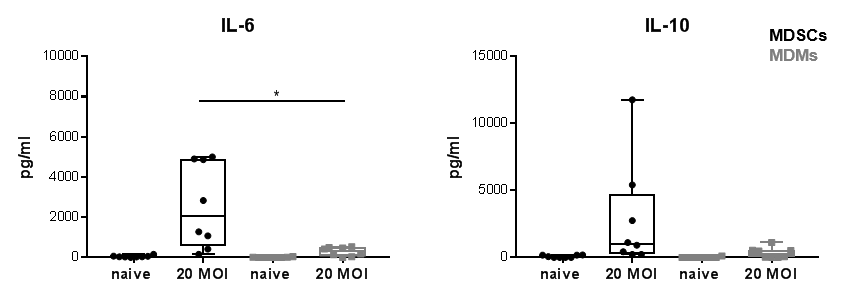


**BCG-infected MDSCs abundantly secrete IL-6 and IL-10.** Concentration of cytokines was determined in cell-free supernatants by ELISA. Each symbol corresponds to a single donor, shown are median±IQR. Data is pooled from 3 independent experiments (n=8 individuals); paired Student’s t-test was done to analyze results, *p≤0.05.

**Supplementary Figure S8**

**Different pathway inhibitors reduced IL-6 and IL-10 secretion from MDSCs or MDMs upon *Mtb* infection.** IL-6 and IL-10 were measured in cell-free culture supernatants from *Mtb*-infected (MOI 10) MDSCs and MDMs by ELISA. Cells were treated with inhibitors at indicated concentrations and supernatants were collected at 24 hr post infection. Each symbol corresponds to a single donor, shown are median±IQR. Data is pooled from 2 independent experiments (n=6 individuals); Wilcoxon test p-value *p≤0.05 and **p≤ 0.01.

**Supplementary Figure S9**

**
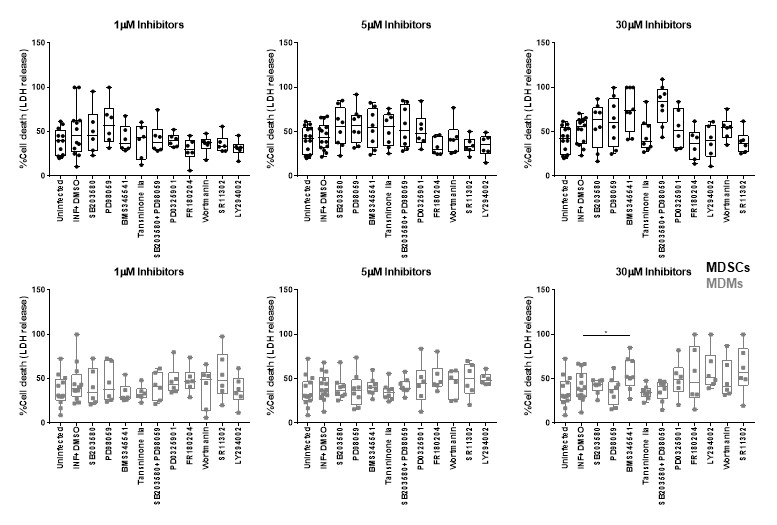
**

**Cell viability is left largely unaltered by chemicals employed to inhibit various signaling pathways.** LDH abundance in cell-free supernatants was measured using commercial available kits. Each symbol corresponds to a single donor, shown are median±IQR; paired Student’s t-test was done to analyze results, *p≤0.05.
